# TRIM24 regulates chromatin remodeling and calcium dynamics in cardiomyocytes

**DOI:** 10.1186/s12964-025-02323-8

**Published:** 2025-07-01

**Authors:** Marco Neu, Anushka Deshpande, Ankush Borlepawar, Elke Hammer, Ahmed Alameldeen, Phillipp Vöcking, Timon Seeger, Michael Hausmann, Norbert Frey, Ashraf Yusuf Rangrez

**Affiliations:** 1 Department of Internal Medicine III (Cardiology and Angiology), University Hospital Heidelberg, Im Neuenheimer Feld 410, Heidelberg, 69120 Germany; 2 German Center of Cardiovascular Research (DZHK), Partnersite Heidelberg/Mannheim, Heidelberg, Germany; 3 Kirchoff-Institute for Physics, University of Heidelberg, Im Neuenheimer Feld 227, Heidelberg, 69120 Germany; 4 Cellular Adaptation and Bioenergetics Group, Institute for Translational Medicine, Medical School Hamburg, Am Kaiserkai 1, Hamburg, 20457 Germany; 5 Interfaculty Institute of Genetics and Functional Genomics, University of Greifswald, Felix-Hausdorff-Straße 8, Greifswald, 17475 Germany; 6 German Center of Cardiovascular Research (DZHK), Partnersite Greifswald, Greifswald, Germany

**Keywords:** E3 ligase, TRIM24, Ubiquitin–proteasome system, Chromatin remodeling, Calcium homeostasis

## Abstract

**Background:**

Cardiomyocyte proteostasis and calcium homeostasis are critical for maintaining cardiac function, with their dysregulation contributing to cardiac hypertrophy and heart failure. The Tripartite Motif Protein 24 (TRIM24), a well-characterized chromatin reader and transcriptional regulator in cancer, has recently emerged as a potential player in cardiac biology. However, its precise role in cardiomyocytes remains unclear. Using molecular, structural and functional approaches, this study investigates the impact of TRIM24 on cardiomyocyte function and gene regulation.

**Methods:**

To dissect the molecular and functional role of TRIM24, we conducted RNA-sequencing (RNA-seq) and chromatin immunoprecipitation sequencing (ChIP-seq) in neonatal rat ventricular cardiomyocytes (NRVCMs) to identify TRIM24-regulated pathways and transcriptional targets. Super-resolution microscopy and proteomics analysis were employed to examine its influence on chromatin organization and calcium-handling protein distribution. Calcium imaging and cardiomyocyte contractility assays were performed in both NRVCMs and human induced pluripotent stem cell-derived cardiomyocytes (iPSC-CMs) to assess functional effects. Additionally, NFAT activity was assessed to investigate its role in TRIM24-mediated hypertrophic signaling.

**Results:**

Through RNA-seq and ChIP-seq, we identified TRIM24 as a bidirectional transcriptional regulator, predominantly acting as a repressor but also exhibiting context-dependent activation of genes involved in e.g. cytoskeletal organization and calcium signaling. ChIP-seq identified TRIM24 binding at the NFATc4 locus, validated by motif analysis, while functional studies revealed that TRIM24 regulates NFATc4 protein levels and activity, enhancing upon overexpression and reducing upon knockdown. Furthermore, TRIM24 overexpression altered the expression and organization of Ryanodine Receptor 2 (RyR2), Sarcoplasmic/endoplasmic Reticulum Ca^2+^ ATPase 2a (SERCA2a), and Calsequestrin 1 (CASQ1), leading to calcium-handling defects. Super-resolution microscopy revealed a loss of chromatin organization and altered clustering of calcium-handling proteins. Despite a reduction in SERCA2a levels, TRIM24 activated the PI3K-AKT/PLN pathway, increasing phospholamban phosphorylation and compensatory calcium reuptake. In functional assays, TRIM24 overexpression increased beating frequency and calcium cycling in both NRVCMs and iPSC-CMs.

**Conclusion:**

Our findings establish TRIM24 as a novel regulator of chromatin remodeling and cardiomyocyte transcription, directly influencing calcium homeostasis and contractility, with potential implications for cardiac disease.

**Supplementary Information:**

The online version contains supplementary material available at 10.1186/s12964-025-02323-8.

## Introduction

The maintenance of functional protein homeostasis, or proteostasis, is a cornerstone of cardiac health [1]. Proteostasis encompasses the intricate balance of protein synthesis, folding, and degradation, orchestrated by a network of chaperones, autophagy pathways, and the ubiquitin–proteasome system [1, 2]. This dynamic equilibrium ensures the removal of aged, misfolded, and damaged proteins, safeguarding cellular integrity and function. Disruption of protein quality control (PQC) mechanisms can trigger a cascade of detrimental events, including loss of sarcomeric and cytoskeletal proteins, impaired ATP synthesis, and aberrant chromatin remodeling, ultimately driving cardiomyocyte senescence and contributing to cardiac pathology [1, 2]. Targeting key regulators of cardiac proteostasis has thus emerged as a promising therapeutic strategy for combating heart disease. Among these regulators, the Tripartite Motif protein 24 (TRIM24) has recently garnered attention as a novel player in cardiac biology, despite its well-documented role in cancer [3–6].

The TRIM family, characterized by the conserved RBCC (RING-B-box-coiled coil) domain, comprises a group of E3 ubiquitin ligases critical for post-translational modifications and cellular signaling [3, 7, 8]. TRIM24, also known as Transcription Intermediary Factor 1α, uniquely harbors a C-terminal tandem plant homeodomain (PHD) and bromodomain, enabling it to recognize unmodified H3K4 and acetylated H3K23ac on histone tails [9–11]. This dual recognition capacity allows TRIM24 to recruit transcription factors such as estrogen receptor α (ERα), driving cellular proliferation and implicating it in oncogenic processes [4, 12–14]. Indeed, TRIM24 overexpression has been linked to poor survival outcomes in cancers such as breast, bladder, and gastric cancer [12, 14–18].

Paradoxically, TRIM24 also exhibits tumor-suppressive functions. For instance, it interacts with the liganded retinoic acid receptor (RAR) to repress its transcriptional activity, and its inactivation in mice leads to RAR dysregulation and hepatocellular carcinoma (HCC) [19–21]. Furthermore, TRIM24 plays a pivotal role in breast tumorigenesis by reprogramming glucose metabolism in human mammary epithelial cells (HMECs) [22]. Its ability to interpret the methylation state of H3K4 also modulates p53-dependent chromatin accessibility, with TRIM24 loss in mouse embryonic stem cells resulting in chromatin decondensation and heightened transcriptional activity under stress conditions [8, 23].

Recently, TRIM24 has emerged as a critical factor in cardiac pathology. Elevated TRIM24 levels have been observed in patients with hypertrophy and dilated cardiomyopathy, suggesting its potential role in cardiac remodeling [24]. Our previous work has demonstrated that TRIM24 protects dysbindin from ubiquitin-mediated degradation by TRIM32, thereby promoting hypertrophic development [24]. These findings, coupled with the known influence of TRIM24 on chromatin structure and its downstream effects on the transcriptome and proteasome, underscore its potential as a key regulator of cardiac proteostasis. Despite these advances, the functional role of TRIM24 in cardiomyocytes remains poorly understood. This study seeks to bridge this gap by elucidating the role of TRIM24 in chromatin remodeling and calcium homeostasis, two critical processes in cardiac physiology. Using RNA sequencing (RNA-seq) and chromatin immunoprecipitation sequencing (ChIP-seq), we reveal TRIM24 as a bidirectional transcriptional regulator that modulates key pathways in lipid biosynthesis, cytoskeletal organization, and calcium ion transport. Functional assays demonstrate that TRIM24 directly influences cardiomyocyte contractility by regulating the expression and spatial organization of calcium-handling proteins, including Ryanodine Receptor 2 (RyR2), sarcoplasmic/endoplasmic reticulum Ca^2+^-ATPase (SERCA2a), and Calsequestrin 1 (CASQ1). Super-resolution microscopy further reveals that TRIM24 overexpression disrupts the structural integrity of calcium-handling machinery, while contractility assays show a TRIM24-dependent increase in beating frequency and contraction velocity. Additionally, TRIM24 regulates NFATc4 activity, linking it to hypertrophic signaling pathways.

By positioning TRIM24 as a pivotal modulator of calcium homeostasis and chromatin structure in cardiomyocytes, this study provides novel insights into its role in cardiac physiology and pathology. These findings not only expand our understanding of the multifaceted functions of TRIM24 but also highlight its potential as a therapeutic target for calcium-dependent cardiac dysfunctions and hypertrophic cardiomyopathies.

## Materials and methods

### Cloning of TRIM24 overexpression and knockdown constructs

The adenoviral constructs for overexpression and knockdown of TRIM24 were generated as described in Borlepawar et al. (2017) [24]. The TRIM24 expression vector plasmid was kindly provided by Dr. Barton from the University of Texas, Houston. To achieve TRIM24 knockdown, synthetic microRNAs were cloned using the BLOCK-iT™ polymerase II miR RNAi Expression vector kit in combination with the Gateway cloning system (Thermo Fisher Scientific, Waltham, MA, USA). Adenoviral constructs encoding the full-length human TRIM24 cDNA were generated with the ViraPower adenoviral kit (Thermo Fisher Scientific), following the manufacturer’s protocol. Specifically, the previously cloned cDNA in the pDONR221 vector was transferred into the pAd/CMV/V5-DEST destination vector. After digestion with the PacI restriction enzyme, the constructs were transfected into HEK293A cells for adenovirus production. Viral titration was assessed by staining infected HEK293A cells with a fluorescent anti-Hexon antibody. An adenovirus encoding β-galactosidase-V5 (Ad-LacZ; Thermo Fisher Scientific) was used as a control.

### Neonatal rat ventricular cardiomyocyte (NRVCM) isolation and culture

NRVCMs were isolated from the left ventricles of 1–2-day-old Wistar rat pups (Charles River, Lyon, France) following the protocol by Borlepawar et al. (2017) [24]. Briefly, ventricles were minced in ADS buffer (120 mmol/L NaCl, 20 mmol/L HEPES, 8 mmol/L NaH₂PO₄, 6 mmol/L glucose, 5 mmol/L KCl, 0.8 mmol/L MgSO₄, pH 7.4) and subjected to five to six enzymatic digestion steps using 0.6 mg/mL pancreatin (Sigma) at 37 °C and 0.5 mg/mL collagenase type II (Worthington, Lakewood, NJ, USA) in sterile ADS buffer. Digestion was stopped by filtering the cell suspension and adding newborn calf serum. Cardiac fibroblasts were removed via Percoll gradient centrifugation (GE Healthcare, Chicago, IL, USA). NRVCMs were cultured in DMEM supplemented with 10% FCS, 2 mM penicillin/streptomycin, and L-glutamine (PAA Laboratories, Pasching, Austria). Adenoviral infection with LacZ/TRIM24 (MOI: 25) for overexpression studies, or mir-Neg/mir-TRIM24 (MOI: 500) for knockdown studies, was performed 24 h post-isolation in serum-free DMEM containing penicillin/streptomycin. Experiment concerning NFAT inhibition, cells were treated with the selective NFAT inhibitor VIVIT (1 µM; Calbiochem) for 24 h prior to cell processing. VIVIT, a cell-permeable peptide, specifically disrupts the interaction between NFAT and calcineurin without affecting calcineurin's phosphatase activity, thereby preventing NFAT nuclear translocation and transcriptional activity. The inhibitor was added directly to the culture medium, and cells were maintained under standard culture conditions during the treatment period. Cells were harvested 72 h post-infection for downstream analyses.

### Differentiation of human induced pluripotent stem cells into cardiomyocytes

Human induced pluripotent stem cell-derived cardiomyocytes (hiPSC-CMs) were obtained from the Stem Cell Core Facility under the supervision of Dr. Timon Seeger. The facility provided cryopreserved hiPSCs in standardized plate formats, ready for downstream applications. For differentiation, a Matrigel-coated 6-well plate was prepared at least 2 h prior to cell seeding. The cryovial containing the hiPSCs was thawed on ice, and during this period, one well of the plate was washed and filled with 1 mL of hE8 medium (Gibco, London, England) supplemented with 1:1000 ROCK inhibitor (Y-27632, Tocris, Bristol, England). An additional 5 mL of the same medium was prepared in a Falcon tube. The thawed cells were transferred to this tube and centrifuged at 300 × g for 3 min. After careful removal of the supernatant, the cell pellet was resuspended in 1 mL of hES medium containing ROCK inhibitor and seeded into the prepared well. Cells were incubated at 37 °C in a humidified incubator with 5% CO₂. hiPSCs were differentiated into cardiomyocytes using a chemically defined protocol [25]. Briefly, on day 0, cells were treated with 3 mL CDM3 medium supplemented with CHIR99021 (CHIR, MedChemExpress, Monmouth Junction, NJ, USA). On day 2, the medium was changed to only CDM3 [25]. From day 3 to day 4, CDM3 medium supplemented with 5 µM IWP2 (Tocris, Bristol, England) was added. From day 5 to 6, cells were maintained in CDM3 alone. On day 7, the medium was changed to B27 supplement with insulin (B27 + ins, Gibco, London, England). From day 10 to 12, the medium was switched to CDM3L lacking glucose for metabolic selection. Cells were subsequently maintained in B27 + ins medium and passaged on day 15 for further use. All experiments were conducted using hiPSC-CMs at 40 days post-differentiation. To quantify cell surface area, 25,000 cells were seeded per well in a 4-well ibidi Slide and subjected to the appropriate treatments. Following fixation with 4% paraformaldehyde (PFA), cells were blocked and stained for α-actinin. Imaging was performed at 20 × magnification using a Leica Mica fluorescence microscope. Cell surface area was measured by generating binary masks in FIJI (ImageJ) based on α-actinin staining.

### Cardiomyocyte contractility assay

NRVCMs were seeded, cultured and treated in 96-well plates (Thermo Fisher Scientific). One day before the CCA measurement, the cells were incubated with tetramethylrhodamine methyl ester (TMRM, 200 nM, Thermo Fisher Scientific) for 30 min at 37 °C. Subsequently, the cells were washed three times and incubated overnight. The CCA measurement was done with a ZEISS Axio Observer microscope (Zeiss, Oberkochen, Germany) by taking 1500 images with a framerate of 50 per second. Data analysis of the image stack from the cardiomyocyte contraction measurement assay was determined by an in-house MATLAB and ImageJ script.

### Immunostaining

NRVCMs were fixed with 4% PFA for 10 min at 37 °C before washing with PBS. For blocking 2.5% BSA diluted in PBS was used for 1 h at RT. Subsequently, the cells were incubated overnight at 4 °C in blocking solution containing the primary antibodies of choice in their respective dilutions. The following day cells were washed five to six times with PBS before staining with the secondary antibodies of choice in their respective dilution overnight at 4 °C. After washing again for five to six times the cells were incubated with DAPI (D1306, Invitrogen, Waltham, MA, USA) diluted 1:10,000 for 1 h at RT. Images were captured by the LSM980 AIRY confocal microscope (Zeiss, Oberkochen, Germany).

### Localization microscopy

The microscope located at the Light Microscopy Facility of the German Cancer Research Center in Heidelberg was built by W. Schaufler. The detailed description of the setup can be found in M. Krufczik et al. [26]. To ensure a steady temperature an enhanced thermo-mechanical stability was provided, which enabled a localization precision of ± 10 nm. For excitation of Alexa 647, the 642 nm laser was set to 0.8 kW cm^−2^ laser intensity in the sample plane and for excitation of Alexa 594, the 561 nm laser was used at an intensity of 1.87 kW cm^−2^. The exposure time for immunofluorescently stained samples was 100 ms per frame and we collected 2000 frames for each cell. A proprietary software package was used to determine super-resolution signal coordinates based on an algorithm that detects brightness differences between consecutive image frames. Dark states persisting for more than two frames were identified, and the barycenter of these signals was computed. The spatial positions of all detected molecules were logged in the "Orte-Matrix,"while signals below a certain intensity threshold were excluded [27].

To analyze the spatial distribution of signals, Ripley’s statistics was applied to calculate and normalize distances between points [28]. For topological characterization, persistent homology analyses were utilized, following the detailed methodology in [29, 30]. These approaches aim to identify key structural patterns in the dataset, which can be mathematically interpreted as a transformation into a corresponding topological space. Two primary parameters were extracted: the number of components, representing the starting points of the analysis, and the number of holes, which correspond to enclosed regions that appear and disappear during the evaluation process. In this approach, each registered point in the Orte-Matrix is conceptually surrounded by an expanding virtual circle. A bar in the analysis begins at the initial point (radius zero, one component) and extends until two circles overlap, at which point the respective two components merge, and one of the bars stops growing. This process continues, sometimes forming larger structures that enclose empty spaces, referred to as "holes."These holes are also represented by bars that start when a hole is formed and end when the circles overlap the hole completely. In this context the frequency of endpoint values of bars represents a measure for the sizes of the holes. A more detailed explanation can be found in the Supplementary Fig. 1.

### Antibodies

Antibodies used for various experiments in this study were as follows:

AKT, rabbit/mono, Cell Signaling, 9272S,WB (1:1000); phospho-AKT, rabbit/mono, Cell Signaling, 244F9, IF (1:1000); Calsequestrin, rabbit/poly, Invitrogen, PA1-913, WB (1:1000); phospho-CamKII, rabbit/mono, Cell Signaling, 12,716, WB (1:1000); Flag-tag, rabbit/mono, Merck, F2555, ChIP (1:1000); GAPDH, rabbit/mono, Cell Signaling, 2118S, WB (1:1000); H3K4me3, rabbit/poly, Sigma, 07–473, IF/WB (1:1000); H3K27ac, rabbit/poly, Abcam, ab4729, IF/WB (1:1000); NFATc4, rabbit/mono, Cell Signaling, 2183S, WB (1:1000); PLN, mouse/mono, Badrilla, A010-14, WB (1:1000); phospho-PLN (Ser16), rabbit/poly, Badrilla, A010-12AP, WB (1:1000); phospho-PLN (Thr17), rabbit/poly, Badrilla,A010-13, WB (1:1000); Ryanodine Receptor 2, rabbit/poly, Biozol, PA040283, IF/WB (1:1000); TRIM24, rabbit/poly, Proteintech, 14,208–1-AP, IF/WB (1:1000); SERCA2a, mouse/mono, Invitrogen, 2A7-A1, IF (1:1000);WB (1:750); Vinculin, rabbit/mono, Cell Signaling, 4650S, WB (1:1000) Flag-tag, rabbit, mono, Merck, ChIP (1:10,000).

### RNA-sequencing

Library preparation, sequencing and data analysis were commercially carried out at Arraystar Inc. Go Beyond RNA (Rockville, MD, USA) as described earlier [31]. Total RNA from each sample was quantified using a NanoDrop ND-1000 spectrophotometer. For library preparation, approximately 2 µg of total RNA was processed as follows: (1) mRNA was enriched using oligo (dT) magnetic beads to remove rRNA, and (2) the RNA-seq library was generated using the KAPA Stranded RNA-Seq Library Prep Kit (Illumina, San Diego, CA, USA), which incorporates dUTP into the second cDNA strand, ensuring strand specificity. The final libraries were assessed for quality using the Agilent 2100 Bioanalyzer (Agilent, Waldbronn, Germany) and quantified by absolute quantification qPCR. For sequencing on the Illumina HiSeq 4000 platform, the indexed libraries were pooled, denatured into single-stranded DNA using NaOH, captured on the flow cell, amplified in situ, and sequenced for 150 cycles per read in a paired-end mode.

Raw sequencing reads were processed using Solexa pipeline v1.8 (Off-Line Base Caller software, v1.8). Quality control checks were performed with FastQC to assess sequence quality (http://www.bioinformatics.babraham.ac.uk/projects/fastqc/). Adapter sequences were trimmed from the reads using cutadapt, followed by alignment to the reference genome with Hisat2. Transcript abundance was quantified using StringTie, and FPKM values for both gene and transcript levels were computed with the R package Ballgown. Differentially expressed genes and transcripts were identified using Ballgown, while novel transcripts were predicted by comparing assembled sequences against the reference annotation. The CPAT tool was employed to evaluate the coding potential of these sequences. Alternative splicing events were analyzed using rMATS, with visualization performed through dedicated plotting tools. Principal Component Analysis (PCA), correlation analysis, hierarchical clustering, gene ontology, pathway enrichment analysis, scatter plots, and volcano plots were conducted using R, Python, or shell scripts for statistical computing and graphical visualization. GO and KEGG analysis were done using shinygo 0.82 (South Dakota State University, U.S.A.) with a FDR cutoff of 0.05. All source data associated with RNA-seq analyses, including figures presented in the main manuscript, are provided as Supplementary Data Table 1.

### Calcium imaging

For ratiometric calcium imaging, dissociated iPSC-CMs were seeded on Matrigel-coated 35 mm Glass bottom dishes (IBL, Gerasdorf, Austria, thickness 1.5 mm). After 4 days, cells were loaded with 2 μM Fura-2AM (Thermo Fisher Scientific, F1221) with 0.02% Pluronic F-127 (Thermo Fisher Scientific, P3000MP) in Tyrode’s solution at 37 °C, followed by washing with Tyrode’s solution. iPSC-CMs were field-stimulated at 0.5 Hz at 37 °C. Single cell Ca2 + imaging was conducted on an Axio Observer 7 microscope with 40 × oil immersion objective (Zeiss, Oberkochen, Germany). Fura-2 was excited at 340 nm and 380 nm wavelength using a Lambda DG- 4 ultra-high-speed wavelength (Sutter Instrument), and the emission of Fura-2 was collected over 510 nm wavelength. Cells were electrically stimulated at a frequency of 0.5 Hz using the MyoPacer Cell Stimulator (Ion Optix, Westwood, MA, USA) to ensure consistent contractile activity.Raw data exported from Zeiss axio observer were analyzed using a custom Python script (https://github.com/GeorgeMcMullen/CalciPy) built specifically to automate processing of ratiometric calcium imaging data.

### Proteomics analysis

Four bioreplicates from independent experiments were analysed for each condition. Protein was extracted in urea/thiourea (8 M/2 M) by multiple freeze–thaw cycles, and collected by centrifugation (60 min, 20 °C, 16,000 g) after nucleic acid fragmentation using a sonication probe. Three µg of protein from each sample was reduced (dithiothreitol) and alkylated (iodoacetamid) before digestion with trypsin at a protein: enzyme ratio of 25:1 (37 °C, 16 h). Peptide mixtures were desalted on C18 material (µZipTip, Merck Millipore, Darmstadt, Germany). Tandem mass spectrometry on a nano-Acquity UPLC Synapt G2Si mass spectrometer configuration (Waters, Milford, MA, USA) was performed as described earlier [32]. The acquired data were analyzed for peptide and protein identification using the program PLGS v3.3 (Waters) against the FASTA database from the NCBI Reference Sequence Database v.03_2019, which contained 66,850 sequences. The workflow search parameters contained trypsin as protease, one missed cleavage, carbamidomethyl for cysteine as fixed and oxidation of methionine as variable modification. The independent identification output by PLGS was imported in ISOQuant 1.8 [33]. The protein quantification was carried out based on the top three peptides (min. intensity 3000) that had no modifications. Bandwidth parameter for non-linear regression method (LOWESS) used for exploring systematic errors during normalization process was set to 0.3 [34]. Quantification of differences between the groups was done on protein areas using Student’s t-test and p < 0.05 was set as significance threshold.

### Protein preparation and immunoblotting

Seventy-two hours after viral transduction, NRVCMs were lysed by performing three freeze–thaw cycles in RIPA buffer (20 mM Tris, 10 mM DTT, 500 mM NaCl, 1% NP-40, 12.5% glycerol) supplemented with protease and phosphatase inhibitors (Roche, Mannheim, Germany). The lysates were subsequently centrifuged at 18,000 × g for 20 min to pellet cellular debris. Protein concentrations in the cleared supernatants were determined using the DC Protein Assay (Bio-Rad Laboratories, San Francisco, CA, USA). Equal amounts of protein were separated by electrophoresis on either 10% SDS-PAGE or commercially available 4–12% gradient gels (Life Technologies, Carlsbad, CA, USA). Proteins were then transferred onto nitrocellulose membranes and blocked for 1 h at room temperature in 5% non-fat dry milk dissolved in 0.1% TBST (Tris-buffered saline with Tween-20). Membranes were incubated overnight at 4 °C with the appropriate primary antibodies, washed four times with TBST, and then probed with HRP-conjugated secondary antibodies (1:10,000 dilution, Cell Signaling, Danvers, MA, USA). Signal detection was carried out using the ECL Select chemiluminescence system (Merck, Darmstadt, Germany), and protein bands were visualized with a ChemiDoc imaging system (Bio-Rad, San Francisco, CA, USA*)*. Quantitative analysis of band intensities was performed using ImageJ/Fiji software (version 1.46).

### RNA isolation and qPCR

Total RNA was extracted from NRVCMs using TRIzol reagent (Invitrogen, Waltham, MA, USA) according to the manufacturer’s protocol. One microgram of DNA-free total RNA was then reverse-transcribed into cDNA using the LunaScript first-strand cDNA synthesis kit (New England Biolabs GmbH, Frankfurt, Germany). Quantitative real-time PCR (q-PCR) was performed using the EXPRESS SYBR Green ER reagent (Life Technologies, Carlsbad, CA, USA) on a LightCycler 480 II (Roche, Mannheim, Germany). The thermal cycling conditions consisted of an initial denaturation at 95 °C for 3 min, followed by 40 cycles of 15 s at 95 °C and 45 s at 60 °C. Expression levels were normalized to Rpl32 as internal reference genes (primer sequences are provided in Supplementary Table 1). All experiments with NRVCMs were performed in six replicates and repeated three times.

### NFATc4 gene reporter assay

NFATc4 reporter gene assay shown in this study was performed in NRVCMs as described previously [35]. Briefly, cells were infected with LacZ Vs TRIM24 adenovirus along with adenovirus Ad-NFATc4-RE-luciferase (MOI: 10) as compared to renilla firefly (MOI: 5) as control (for normalization of measurements). The assay was performed using a dual-luciferase reporter assay kit (Promega, Madison, WI, USA), according to the manufacturer’s guidelines. Luciferase activity was measured photometrically on a Spark Multimode Microplate Reader (Tecan, Männedorf, Switzerland).

### Chromatin immunoprecipitation

Neonatal cardiomyocytes were washed twice with PBS and fixed with 1% (v/v) formaldehyde for 10 min on ice. Crosslinking was quenched by adding glycine to a final concentration of 250 mM for 5 min then snap-frozen. To extract the chromatin, the cells were treated with two different chemical lysis buffers (Lysis Buffer 1: 50 mM Hepes–KOH pH 7.5; 140 mM NaCl; 1 mM EDTA 0.2 ml; 10% glycerol; 0.5% NP-40; 0.25% Triton X-100; Lysis Buffer 2: 200 mM NaCl; 1 mM EDTA (pH 8); 0.5 mM EGTA (pH 8); 10 mM Tris pH 8) while being rotated in each buffer for 10 min at 4 °C. After that the lysates were transferred to the third lysis buffer (50 mM Tris pH 8; 0.1% SDS; 0.95% NP40; 0.1% Na-deoxycholate; 10 mM EDTA; 150 mM NaCl) and sonicated in polymethylpentene tubes (TPX, Diagenode, Seraing, Belgium) for 40 sonication cycles using an automated sonication system (Bioruptor, Diagenode). Sonication cycles were performed at the high energy levels in intervals of 30 s sonication followed by 30 s pause. The immunoprecipitation was done by using a semi-automated IP-Star system (Diagenode) following the manufacture´s guidelines. IP buffers and magnetic beads for precipitation were purchased at Diagenode. For pulldown, we used 1 μg of chromatin for each sample and the above-mentioned antibody anti-FLAG tag. The chromatin samples were subjected to RNAse (Qiagen, Venlo, Netherlands) and Proteinase K treatment (Roche, Mannheim, Germany). Reverse crosslinking was performed at 65 °C overnight. After using Qubit® dsDNA HS assay kit (Thermo Fisher Scientific) to measure the chromatin concentrations, the DNA was ethanol precipitated for quality control by agarose gel electrophoresis. The preparation of the libraries was done using the NEBNext DNA Library Prep Master Mix Set for Illumina (E6040) according to manufacturer´s protocol (ChIPseq data: E-MTAB-8011). The Sequencing was performed by Novogene (Cambridge, England*)*. The analysis was done using shinygo 0.82 with an FDR cutoff of 0.05.

### Statistical analysis

All results are shown as the means ± SEM. The statistical analyses of the data were performed using a two-tailed Student’s *t-*test. p-values of less than 0.05 were considered statistically significant.

## Results

### TRIM24 alters cardiomyocyte transcriptome

**Fig. 1:**
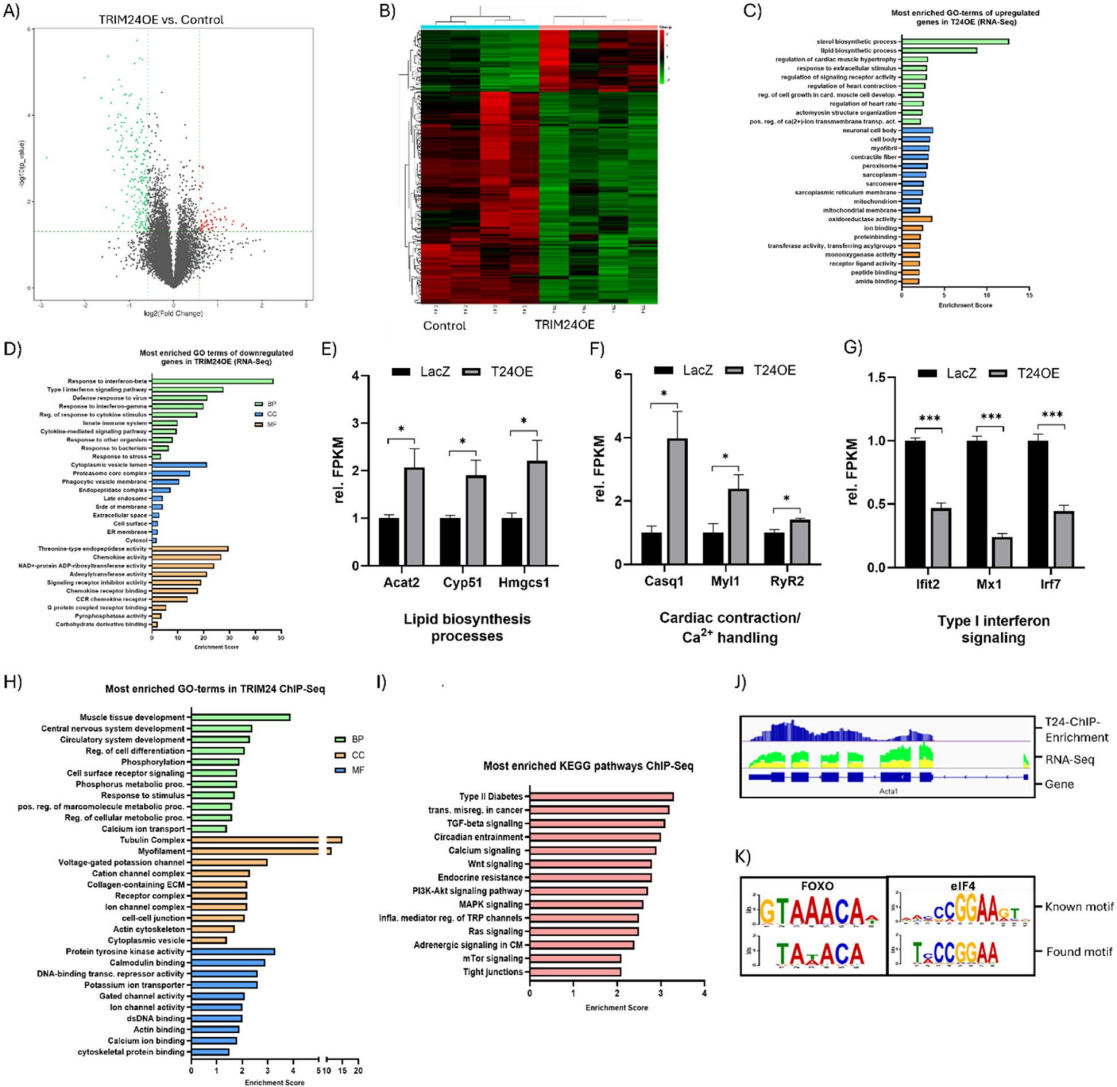
TRIM24 interacts with transcription factors and modulates cardiac gene regulation. Volcano Plot (**A**) and a heat map (**B**) indicating the downregulated (green) or upregulated (red) genes upon TRIM24 overexpression (T24OE) in neonatal rat ventricular cardiomyocytes (NRVCMs). **C** Most enriched GO-terms of upregulated genes in TRIM24OE. **D** Most enriched GO-terms of downregulated genes in TRIM24OE. Bar graphs indicating the selection of upregulated genes due to TRIM24OE suggesting its potential role in lipid biosynthesis (**E**), cardiac contraction and calcium homeostasis (**F**) and in the immune system (**G**). **H** Most enriched GO-terms of enriched peaks identified by Chromatin Immunoprecipitation-sequencing (ChIP-seq) in neonatal rat ventricular cardiomyocytes with TRIM24 overexpression. **I** Most enriched KEGG pathways from ChIP-seq data. **J** Representative examples of enriched ChIP peaks of TRIM24 in comparison with RNA-Seq readouts. Blue: TRIM24 enrichment reads; Red: background reads of Input; green: RNA-Seq reads of TRIM24 overexpression; yellow: RNA-Seq reads of Control. **K** Examples of identified potential TRIM24 binding motifs from the ChIP-seq data. For both RNA-seq and ChIP-seq analyses, four biological replicates were used, and results were validated independently by qPCR. Statistical significance was determined using two-tailed Student's *t* test. *Error bars* show means ± SEM. *, *p* < 0.05; **, ***, *p* < 0.001

TRIM24 has been extensively studied in the context of cancer, where it exhibits diverse and sometimes opposing roles in cellular proteostasis, acting as a transcriptional co-regulator depending on the cellular context. However, its impact on the cardiac transcriptome remains largely unexplored. To address this gap, we performed RNA sequencing (RNA-Seq) on neonatal rat ventricular cardiomyocytes (NRVCMs) overexpressing TRIM24 (TRIM24OE). This analysis revealed significant alterations in the transcriptomic landscape, with 266 genes differentially expressed in TRIM24OE cells compared to controls (Fig. 1A, B). Among these, 206 genes were downregulated, and 60 were upregulated, suggesting that TRIM24 primarily functions as a transcriptional repressor in cardiomyocytes, albeit with context-dependent co-activator activity.

To elucidate the biological significance of these transcriptional changes, we conducted a Gene Set Enrichment Analysis (GSEA) (Supplementary Fig. 2 A, B) and Gene Ontology (GO) analysis, including GO-Biological Process (BP), GO-Cellular Component (CC), and GO-Molecular Function (MF) (Fig. 1C, D). The upregulated genes e.g. acyl-CoA:cholesterol acyltransferase 2 (*Acat2*) (~ twofold with p = 0.037), cytochrome P450, family 51 (*Cyp51*) (~ 1.9 fold with p = 0.031), 3-Hydroxy-3-Methylglutaryl-CoA Synthase 1 (*Hmgcs1*) (~ 2.2 fold with p = 0.034), were significantly enriched in pathways related to lipid and sterol biosynthesis (GO-BP: Lipid biosynthetic process; GO:0008203) (Fig. 1E, Supplementary Fig. 2 A). Notably, pathways associated with calcium homeostasis (GO:1,904,427) and cardiac contraction (GO:0002027; GO:0008016) were also prominently upregulated, including genes such as *Casq1* (~ 4.0 fold with p = 0.010), Myosin light chain 1 (*Myl1*) (~ 2.4 fold with p = 0.039), and *RyR2* (~ 1.4 fold with p = 0.020) (Fig. 1F, Supplementary Fig. 2 A). Furthermore, the analysis revealed substantial changes in the organization of the cardiac cytoskeleton, particularly in actomyosin structure organization (GO:0031032), sarcomere assembly (GO:0030017), and contractile fibers (GO:0043292) (Supplementary Fig. 2 A). Additionally, mitochondria-related genes (GO:0005739; GO:0031966) were upregulated, indicating a potential influence of TRIM24 on mitochondrial metabolism (Supplementary Fig. 2 A). In contrast, the downregulated genes such as Interferon-induced protein with tetratricopeptide repeats 2 (*Ifit2*) (~ 0.46 fold with p = 2.82152E-05), MX Dynamin Like GTPase 1 (*Mx1*) (~ 0.24 fold with p = 2.98812E-06), Interferon-regulatory factor 7 (*Irf7*) (~ 0.44 fold with p = 0.000221), were predominantly associated with immune response pathways, including interferon-α and interferon-γ signaling (GO:0006955; GO:0034097) (Fig. 1D, G, Supplementary Fig. 2B). GSEA further indicated a downregulation of genes involved in mitochondrial function (Supplementary Fig. 2B), suggesting a complex role for TRIM24 in balancing metabolic and immune responses in cardiomyocytes. These findings collectively demonstrate that TRIM24 is likely a key regulator of diverse biological processes in cardiomyocytes, including lipid biosynthesis, cytoskeletal organization, calcium homeostasis, and mitochondrial metabolism.

### TRIM24 functions as a transcriptional coregulator in cardiomyocytes

Although TRIM24 lacks intrinsic DNA-binding capability, its conserved domains enable it to function as a transcriptional co-regulator, modulating nuclear receptor activity in both a positive and a negative manner. To investigate whether the transcriptional changes observed in our RNA-seq data result from the interactions of TRIM24 with other transcription factors, we performed chromatin immunoprecipitation sequencing (ChIP-Seq) in cardiomyocytes. Using Discriminative Regular Expression Motif Elicitation (DREME), we identified short motifs highly enriched in TRIM24 pull-down sequences. Comparison of these motifs against known databases using the Tomtom tool revealed potential TRIM24 binding partners, including established interactors like RaRα, as well as novel candidates from the eIF, STAT, and FOXO families (Supplementary Table 2).

Gene Ontology (GO) and KEGG pathway enrichment analyses of TRIM24 binding sites revealed significant overlaps with RNA-seq findings, including pathways related to calcium channel activity (RNO04020), cell adhesion, metabolic processes (particularly macromolecule and lipid metabolism), and inflammatory responses (Fig. 1H, I). Pathways uniquely identified in ChIP-seq included DNA binding, protein binding, and cell–cell recognition. KEGG analysis further implicated TRIM24 in cancer-related pathways, Type II diabetes, adrenergic signaling (RNO04261), and PI3K-AKT signaling (Fig. 1I). Notably, enriched pathways such as FOXO signaling (RNO04068) and circadian entrainment (RNO04713) align with motif findings for FOXO1, FOXO3, eIF2, and eIF4 (Fig. 1J, K). Of particular relevance to cardiac biology, muscle contraction pathways, including actin cytoskeleton regulation (RNO04810), cardiac muscle contraction (RNO04260), and calcium signaling, were significantly enriched, corroborating the RNA-seq data and underscoring the role of TRIM24 in calcium-dependent cardiac function.

TRIM24 enrichment peaks exhibited diverse patterns, ranging from sharp, high-intensity peaks to broad, diffused accumulations spanning kilobases (Supplementary Fig. 3A-F). These patterns suggest distinct functional roles for TRIM24, from direct transcriptional regulation to broader chromatin interactions. Genomic distribution analysis revealed no preferential binding to specific chromosomes or loci, with notably low enrichment on chromosome X (Supplementary Fig. 3G). TRIM24 binding sites were distributed across intergenic and gene regions, with marked enrichment in regions 500 base pairs upstream to 2 kilobases (TSS500–TSS2K) relative to transcription start sites (TSS) (Supplementary Fig. 3H). However, low binding frequencies in TSS100 and TSS200 intervals indicate that TRIM24 does not primarily bind directly at promoters. Enrichment was also observed in distal upstream (U10K–U20K) and downstream (D10K–D20K) regions, as well as within gene bodies and downstream of transcribed regions (D2K–10 K and TTS500–2K). For example, genes such as *Actb* and *Actc* exhibited broad TRIM24 distribution across gene bodies, while *Acox2* and *Dpf1* displayed sharp, high peaks within introns or exons. Notably, the *Trim24* gene itself showed sharp enrichment within an exon. Downstream enrichment was observed in genes like *Tpm1*, *Bmpr1a*, and the mitochondrial regulator *Tfam*, typically characterized by sharp peaks.

These findings highlight TRIM24 likely acts as a versatile transcriptional co-regulator in cardiomyocytes, orchestrating gene expression through interactions with diverse transcription factors and chromatin remodeling, ultimately influencing pathways critical for cardiac function and disease.

### Alterations in TRIM24 levels significantly affect the state of chromatin landscape in cardiomyocytes

**Fig. 2:**
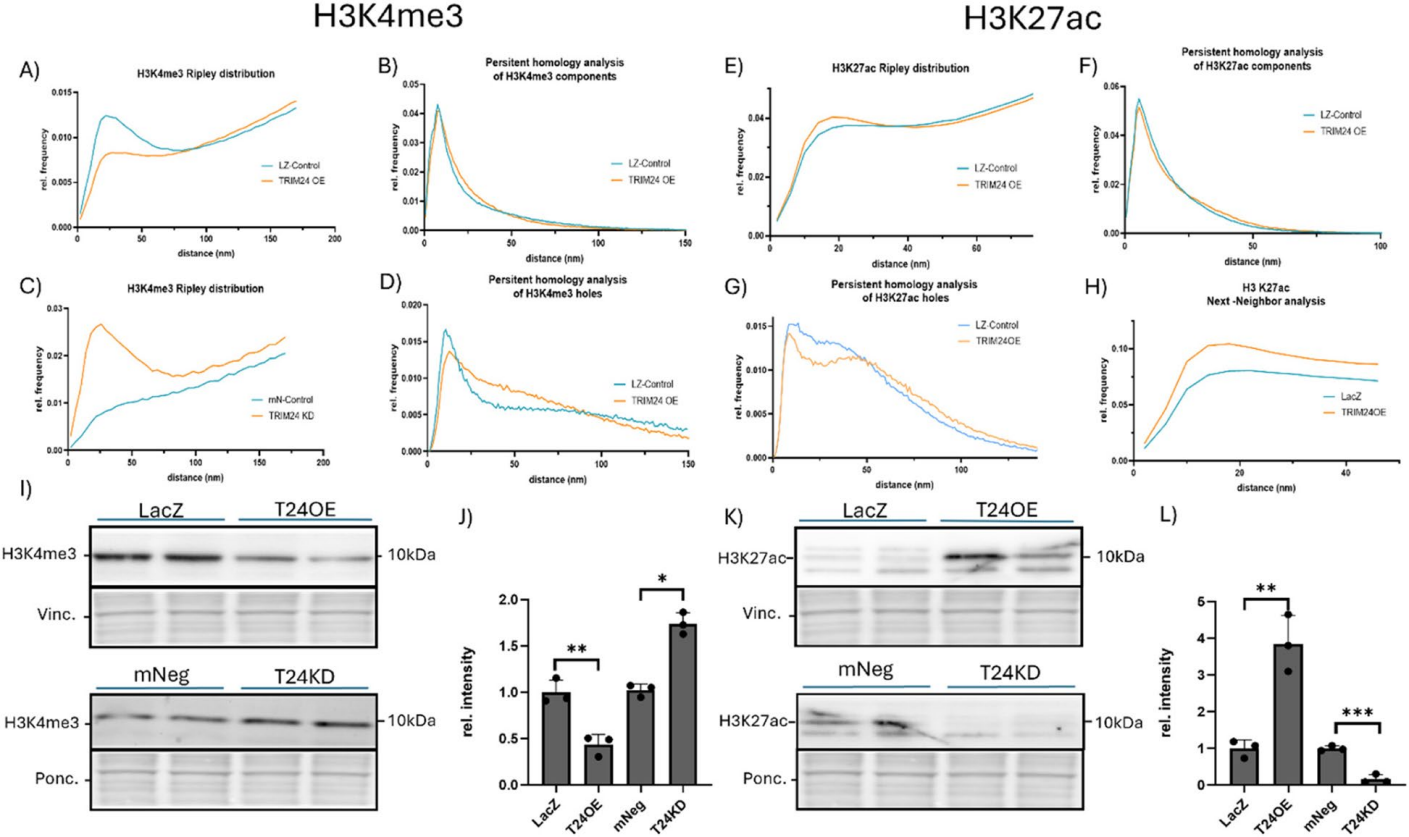
Alterations in TRIM24 levels significantly affect the state of chromatin landscape in cardiomyocytes. Results from the super-resolution localization microscopy are depicted as (**A**) Comparison between the H3K4me3-Ripley analysis of LacZ-Control and TRIM24 overexpression, (**B**) Persistent homology analysis of H3K4me3 components in LZ-Control and TRIM24 overexpression, (**C**) Comparison between the H3K4me3-Ripley analysis of mNeg-Control and TRIM24 knockdown, (**D**) Persistent homology analysis of H3K4me3 holes in LZ-Control and TRIM24 overexpression, (**E**) Comparison between the H3K27ac-Ripley analysis of LacZ-Control and TRIM24 overexpression, (**F**) Persistent homology analysis of H3K27ac holes in LZ-Control and TRIM24 overexpression. (**G**) Persistent homology analysis of H3K27ac components in LZ-Control and TRIM24 overexpression, (**H**) Comparison between the H3K27ac-Next-Neighbor-Analysis of LacZ-Control and TRIM24 overexpression, Immunoblots indicating the levels of H3K4Me3 (**I**), and H3K27ac (**K**) upon TRIM24 overexpression or knockdown. The densitometry of respective blots is shown in (**J**) and (**L**). Statistical significance was determined using two-tailed Student's *t* test. *Error bars* show means ± SEM. *, *p* < 0.05; **, *p* < 0.01; ***, *p* < 0.001. Localization microscopy analyses were based on two independent experiments performed in triplicates, comprising a total of 60 cells per condition per experiment. For immunoblotting, data were generated from three independent experiments, each including three biological replicates per condition

Our results from the ChIP-Seq and RNA-seq data analyses potentially suggest TRIM24 as a transcriptional co-regulator and bidirectional (both suppressor and activator) modulator of cardiomyocyte transcription. This leads to the question of the mechanism by which TRIM24 exerts its suppressive or inductive influence on transcriptional activity. Previous research has identified TRIM24 as a key regulator of gene expression by modulating chromatin states through interactions with modified histones via its PHD and bromodomains. Thus, we stained histone modifications H3K4me3 and H3K27ac to further investigate the mechanisms underlying this transcriptomic regulation, changes in chromatin organization in relation to TRIM24 levels using localization microscopy. As a marker of active promoters, H3K4me3 exhibited significant structural changes under TRIM24 overexpression. Our Ripley’s L-analysis revealed a decreased maximum, indicating a loss of organizational integrity due to reduced H3K4me3 clustering (Fig. 2A). Persistent homology analysis of cluster components (dimension 0) showed only minor deviations, whereas analysis of holes (dimension 1) displayed a distinct shift: While the frequency of holes at smaller distances decreased, holes at a distance from 25 to 75 nm are more frequent, suggesting an increased spacing between H3K4me3 clusters and more prominent structural gaps (Fig. 2B, D). Conversely, TRIM24 knockdown resulted in an increased Ripley maximum, suggesting greater H3K4me3 cluster organization (Fig. 2C, Supplementary Fig. 4). These findings suggest that chromatin relaxation upon TRIM24 knockdown enhances transcriptional activity (the accessibility of DNA is higher in relaxed chromatin than in compacted), whereas TRIM24 overexpression induces chromatin compaction, repressing transcription.

Our Ripley’s L-analysis revealed a significant increase in the maximum under TRIM24 overexpression, indicating higher structure organization (clustering) of H3K27ac (Fig. 2E). Persistent homology analysis of cluster components showed no significant differences, but hole analysis shows an overall reduction in frequency of holes (Fig. 2F, G). To further validate this observation, we performed nearest-neighbor distance analysis (NNA), which increased maxima confirmed increased density within H3K27ac clusters under TRIM24 overexpression, reinforcing its role in transcriptional activation (Fig. 2H). Furthermore, immunoblotting analyses demonstrated decreased H3K4me3 and increased H3K27ac levels in TRIM24-overexpressing cells, whereas, TRIM24 knockdown exhibited opposite results, further strengthening the localization microscopy findings (Fig. 2I-L).

Together, our findings link the transcriptional suppression due to TRIM24 overexpression to chromatin compaction (H3K4me3) and its expression-promoting effect to enhanced H3K27ac clustering. This further highlights TRIM24 as a key and bidirectional modulator of chromatin structure and transcriptional regulation in cardiomyocytes.

### Proteomics analysis revealed TRIM24 to be associated with hypertrophic pathways and Calcium homeostasis

**Fig. 3:**
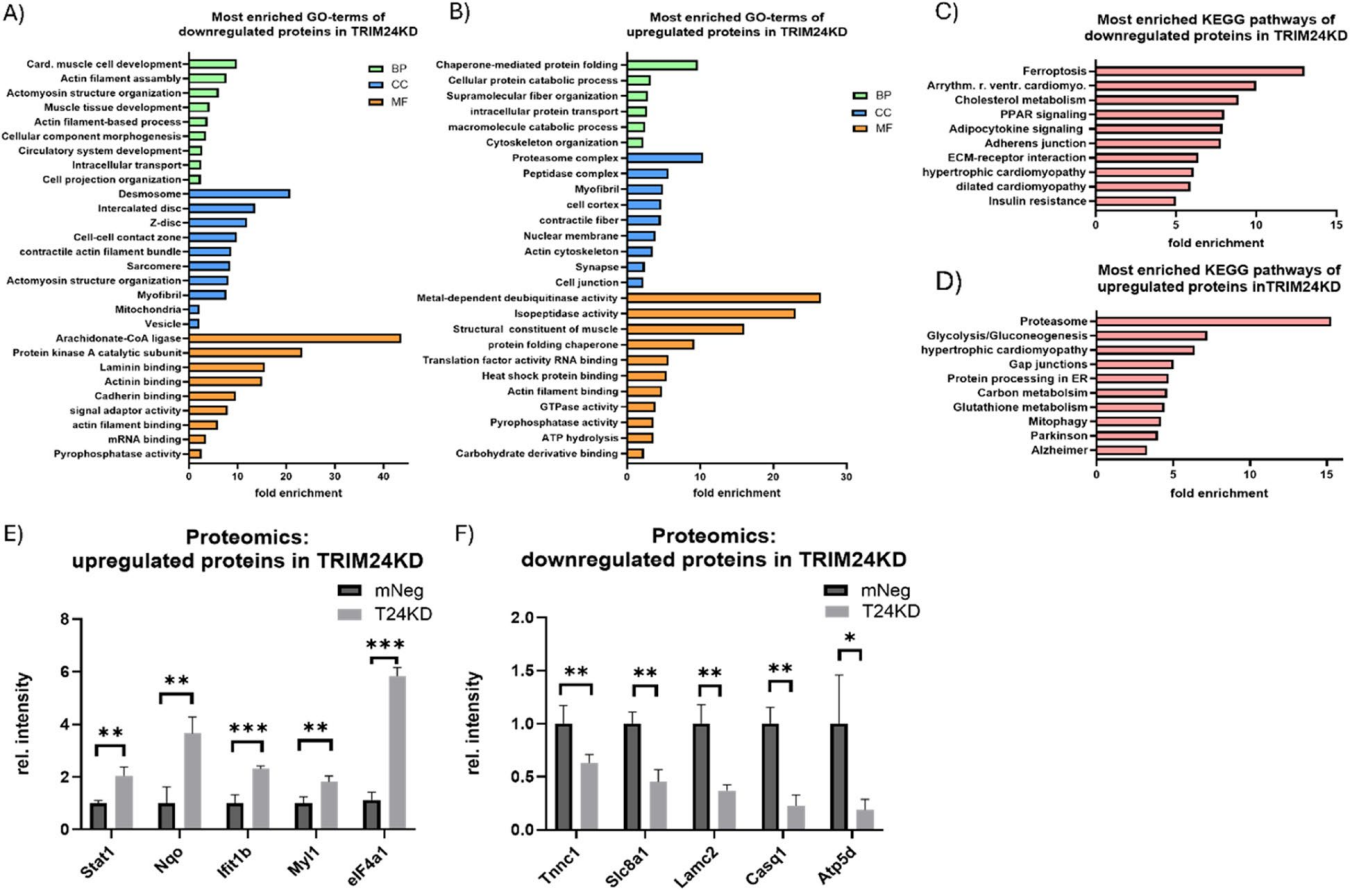
Proteomics analysis revealed TRIM24 to be associated with hypertrophic pathways and Calcium homeostasis. Most enriched gene-ontology (GO) terms for the downregulated (**A**) and upregulated (**B**) proteins in NRVCMs after TRIM24 knockdown (TRIM24KD) compared to the control (mNeg). Most enriched KEGG pathways for the downregulated (**C**) and upregulated (**D**) proteins in NRVCMs upon TRIM24KD**.** Bar graphs indicate some of the selected upregulated (**E**) or the downregulated (**F**) proteins from the proteomics findings. Statistical significance was determined using two-tailed Student's *t* test. *Error bars* show means ± SEM. *, *p* < 0.05; **, *p* < 0.01; ***, *p* < 0.001

Given that the transcriptomics and ChIP-seq data revealed a bidirectional role for TRIM24 as a transcriptional regulatorwe further investigated how its depletion affects proteostasis by performing a proteomics analysis of TRIM24 knockdown in NRVCMs. GO analysis of downregulated proteins highlighted the involvement of TRIM24 in cytoskeletal organization, contractility, and cell–cell connections (Fig. 3A). KEGG analysis revealed significant enrichment in cholesterol metabolism (Rno04979), PPAR (Rno03320), and insulin signaling (Rno04931) (Fig. 3C), consistent with their upregulation in TRIM24-overexpressing cells. Additionally, key components of hypertrophic and dilated cardiomyopathy pathways (Rno05410; Rno05414), including ITGA, NCX, and TnC, were downregulated (Fig. 3C). Interestingly, KEGG analysis of upregulated proteins also implicated hypertrophic and dilated cardiomyopathy pathways, particularly through Laminin, α- and β-Dag, and ACTG1, suggesting opposing or compensatory regulatory mechanisms (Fig. 3D). Other enriched pathways included glutathione metabolism (Rno00480), mitophagy (Rno04137), and adrenergic signaling, with GO analysis indicating partial upregulation of cytoskeletal pathways, further supporting the compensatory hypothesis (Fig. 3B).

Of note, several proteins upregulated in NRVCMs upon TRIM24KD were found to be downregulated in the RNA-Seq of TRIM24OE cells, including immune-related factors like STAT1 and IFIT1b (Fig. 3E, Supplementary Data Table 1). Additionally, the upregulation of eIF4 in TRIM24KD cells aligns with ChIP-Seq findings, as it shares transcription motifs with TRIM24 (Fig. 3E). Furthermore, some of the important proteins such as Troponin C1, Calsequestrin 1, ATP synthase, etc. were significantly downregulated (Fig. 3F). T24OE on the other hand resulted in upregulation of metabolic pathways such as glycolysis, protein translation and eIF2 signaling, whereas extracellular matrix receptor and cell–matrix adhesion were significantly downregulated (Supplementary Fig. 5A-F). These results reinforce the critical role TRIM24 plays in maintaining proteostasis and regulating pathways essential for cardiac function.

### TRIM24 alters cardiomyocyte contractility by regulating the levels and organization of CASQ1, RyR2, and SERCA2a

**Fig. 4:**
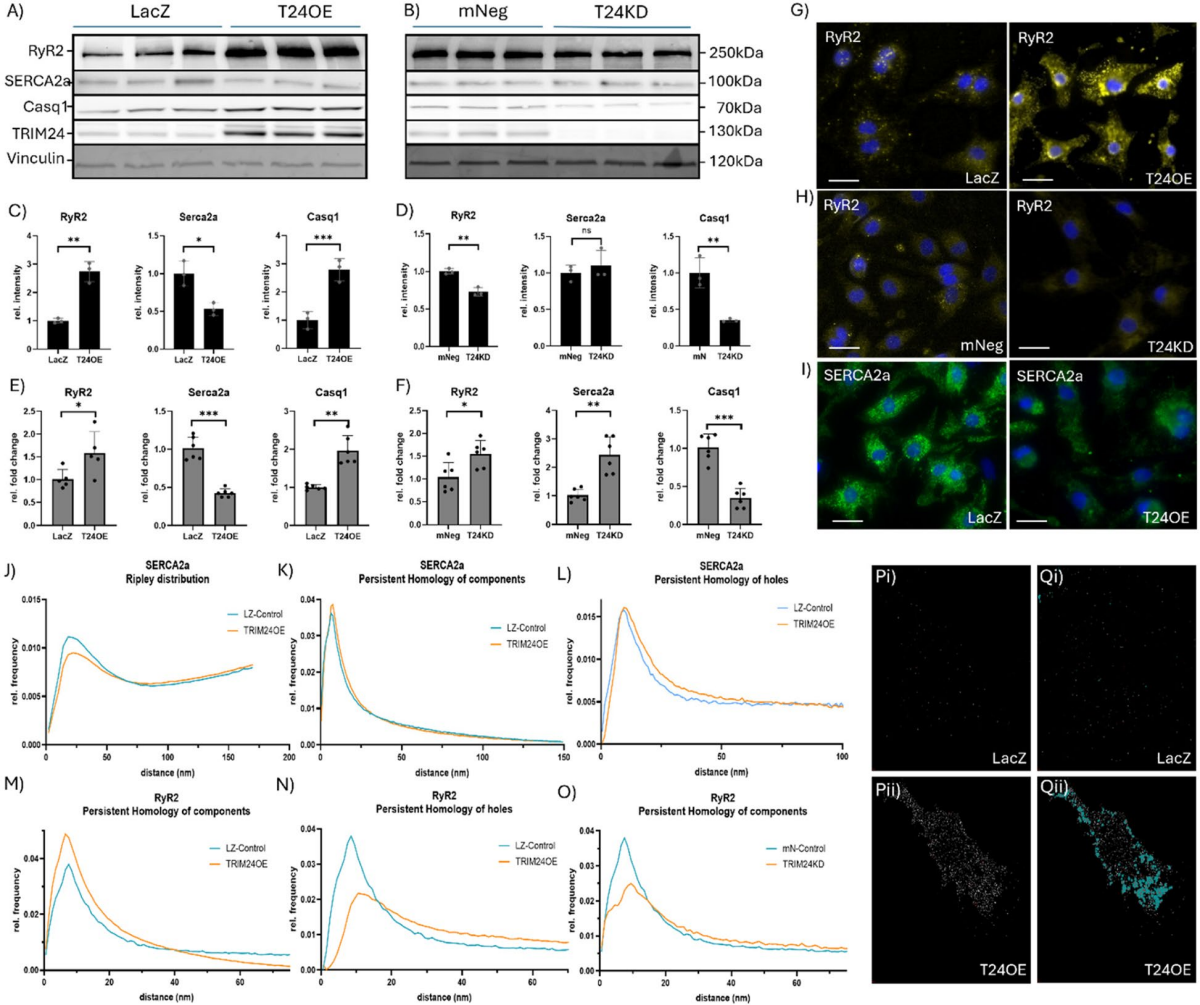
TRIM24 alters calcium homeostasis by regulating the levels and organization of CASQ1, RyR2, and SERCA2a. Immunoblots indicating the protein levels of RyR2, CASQ1, and SERCA2a upon TRIM24 overexpression (**A**) or knockdown (**B**), and their densitometry is presented in (**C**) and (**D**), respectively. Bar graphs indicating the transcript levels of RyR2, CASQ1, and SERCA2A upon TRIM24 overexpression (**E**) or knockdown (**F**), determined by qPCR. Immunofluorescence microscopy images indicating the levels of RyR2 when TRIM24 is overexpressed (**G**) or knocked down (**H**), and SERCA2A when TRIM24 is overexpressed (**I**). **J** Ripley’s L-analysis of SERCA2a in TRIM24 overexpression (T24OE) Overexpression of TRIM24 causes a loss of organization structure. The Persistent Homology analysis of the components (**K**) showed no significant alterations, but the analysis of the holes (**L**) showed a deviation towards larger holes suggesting less dense cluster towards the edge. The persistent homology of RyR2 components shows a significant increase in cluster number and cluster size (**M**). Since there are more receptors, holes are less likely to be generated as to be seen in (**N**). Conversely, in TRIM24 knockdown (T24KD) the organization structure of RyR2 significantly diminishes, causing clusters to not form correctly (**O**). Localization image of RyR2 signals in Control (**Pi**) and T24OE (**Pii**). Cluster closing function of RyR2 signals in Control (**Qi**) and T24OE (**Qii**). Statistical significance was determined using two-tailed Student's *t* test. *Error bars* show means ± SEM. *, *p* < 0.05; **, *p* < 0.01; ***, *p* < 0.001; *ns*, non-significant. Localization microscopy analyses were based on three independent experiments performed in triplicates, comprising a total of 60 cells per condition per experiment. For immunoblotting, data were generated from three independent experiments, each including three biological replicates per condition. Whereas qPCR experiments were carried out in three independent experiments with six biological replicates each

Our findings from transcriptomics, proteomics and ChIP-seq reveal that TRIM24 likely plays a crucial role in cardiomyocyte contractility by modulating calcium homeostasis through its regulation of key calcium-handling proteins, including RyR2, SERCA2a, and CASQ1. To further validate these findings and elucidate the molecular basis of these effects, we examined the expression and spatial organization of RyR2, SERCA2a, and CASQ1. qPCR and Western blot analyses revealed that TRIM24 overexpression increased RyR2 and CASQ1 while reducing SERCA2a expression, at both transcript and protein levels (Fig. 4A, C, E). In contrast, TRIM24 knockdown led to a decrease in CASQ1 mRNA levels but an upregulation of RyR2 and SERCA2a (Fig. 4F). At the protein level, CASQ1 levels followed the transcriptional pattern, showing a significant increase in TRIM24-overexpressing cells and a decrease in knockdown cells (Fig. 4A-D). RyR2 protein levels were elevated in TRIM24-overexpressing NRVCMs but, in contrast to the qPCR results, were reduced in TRIM24 knockdown cells (Fig. 4A-D). SERCA2a protein levels were downregulated in TRIM24-overexpressing cells and remained unchanged in knockdown conditions (Fig. 4A-D). Immunostaining of RyR2 further confirmed these findings, revealing an increase in signal intensity and the number of foci in TRIM24-overexpressing cells, whereas knockdown resulted in a striking reduction in both parameters (Fig. 4G, H). Similarly, SERCA2a staining showed decreased intensity upon TRIM24 overexpression (Fig. 4I).

Beyond expression changes, TRIM24 also modulated the structural organization of RyR2 and SERCA2a clusters, a critical factor for maintaining calcium flux and cardiomyocyte function. Super-resolution microscopy coupled with Ripley’s L-analysis revealed that TRIM24 overexpression disrupted SERCA2a cluster integrity, decreasing structural organization, as evidenced by a decrease in the peak maximum of the Ripley frequency curve. Persistent homology analysis shows a higher frequency of topological holes at greater distances. (Fig. 4J-L). The RyR2 organization was even more profoundly affected, with overexpression leading to such excessive protein accumulation that Ripley’s L-analysis became unfeasible (Fig. 4M-N, P-Q, Supplementary Fig. 6A-B). Persistent homology analysis of the components exhibits an increase in maximum as well as an increase in width, indicating an increase in cluster size and density. However, while the increased density is reflected by a decrease in frequency of holes at smaller distances, a loss of structural integrity at the edges, with a significantly higher frequency of holes at greater distances is evident (Fig. 4M, N, P-Q). In contrast, TRIM24 knockdown impaired RyR2 clustering capacity, as indicated by a reduction in the Ripley’s L-maximum and a profound structural de-organization observed in persistent homology analysis (Fig. 4O, Supplementary Fig. 6C-D).

**Fig. 5:**
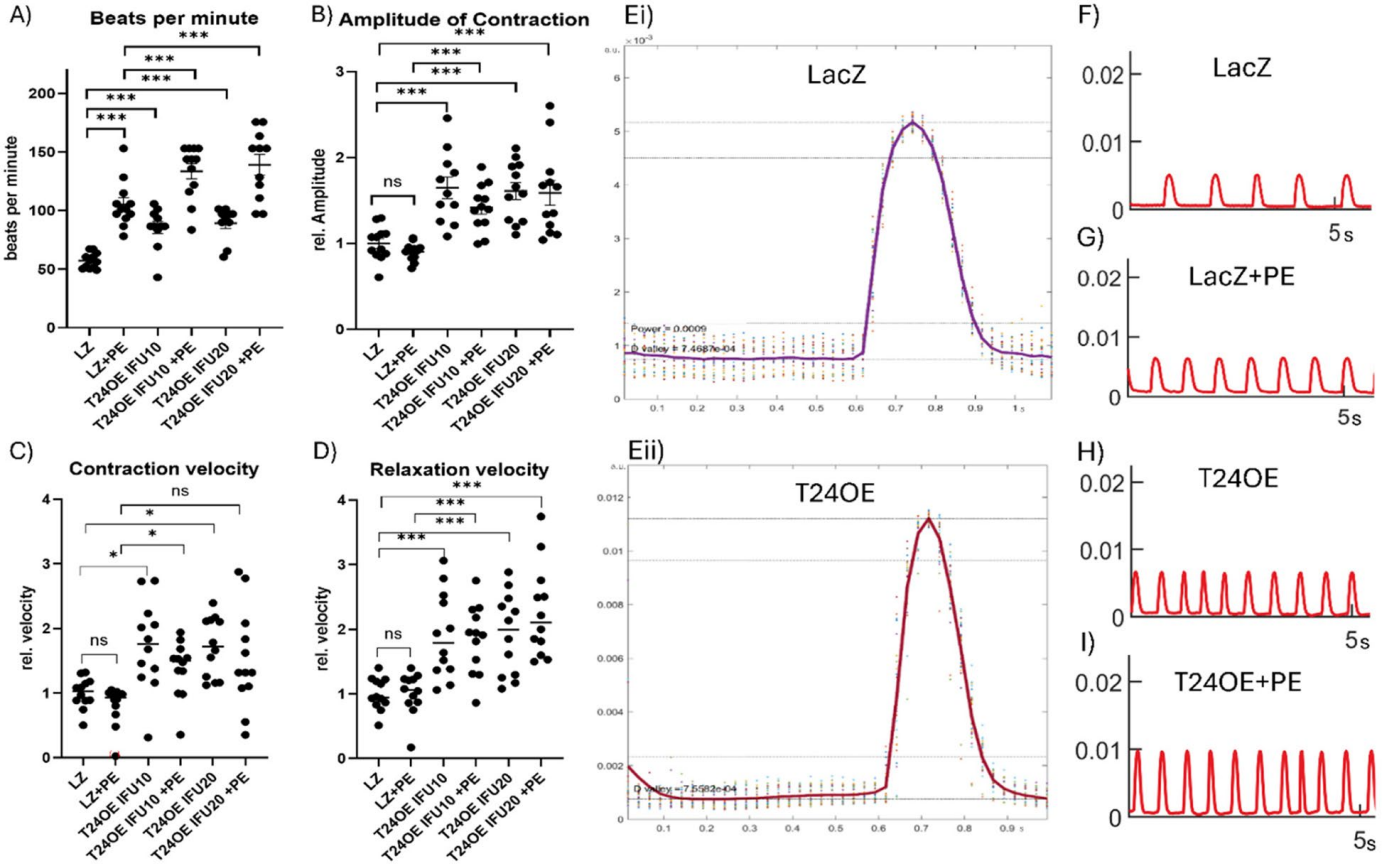
Cardiomyocyte contractility assay reveals enhanced contractile performance and beating frequency upon TRIM24 overexpression. Representative contraction traces and quantitative analysis show that TRIM24-overexpressing cardiomyocytes display increased beating frequency (**A**, **F-I**), contraction amplitude (**B**, **E**), maximal contraction velocity (**C**), and relaxation velocity (**D**), both under baseline conditions and upon phenylephrine (PE) stimulation, compared to respective LacZ control. Panel E shows a representative contraction trace from a single technical replicate; the x-axis represents time (s), and the y-axis shows contraction in arbitrary units (a.u.), derived from relative motion within the field of view. CCA was performed in three independent experiments with 12 biological replicates each

We then determined the functional consequences of these molecular and structural changes in cardiomyocytes using contractility assays. TRIM24-overexpressing NRVCMs exhibited a significant increase in beating frequency and contraction amplitude, both under baseline conditions and upon stress due to phenylephrine (PE) treatment (Fig. 5A-B, E-I). Contraction and relaxation velocities were also enhanced, as evidenced by differentiation of contraction curves, which revealed a broader distribution of contraction and relaxation speeds in TRIM24-overexpressing cells (Fig. 5C-E). In contrast, TRIM24 knockdown resulted in severe functional impairment, with cells displaying highly irregular and erratic contractions, precluding quantitative assessment. Collectively, these findings establish TRIM24 as a key regulator of calcium homeostasis and cardiomyocyte contractility by modulating both the levels and spatial organization of CASQ1, RyR2, and SERCA2a. By influencing the structural integrity of calcium-handling protein clusters, TRIM24 governs the efficiency of calcium-induced calcium release, ultimately dictating the contractile behavior of cardiomyocytes.

### *TRIM24 modulates cellular homeostasis and hypertrophy *via* NFATc4 and PI3K-AKT pathways*

**Fig. 6:**
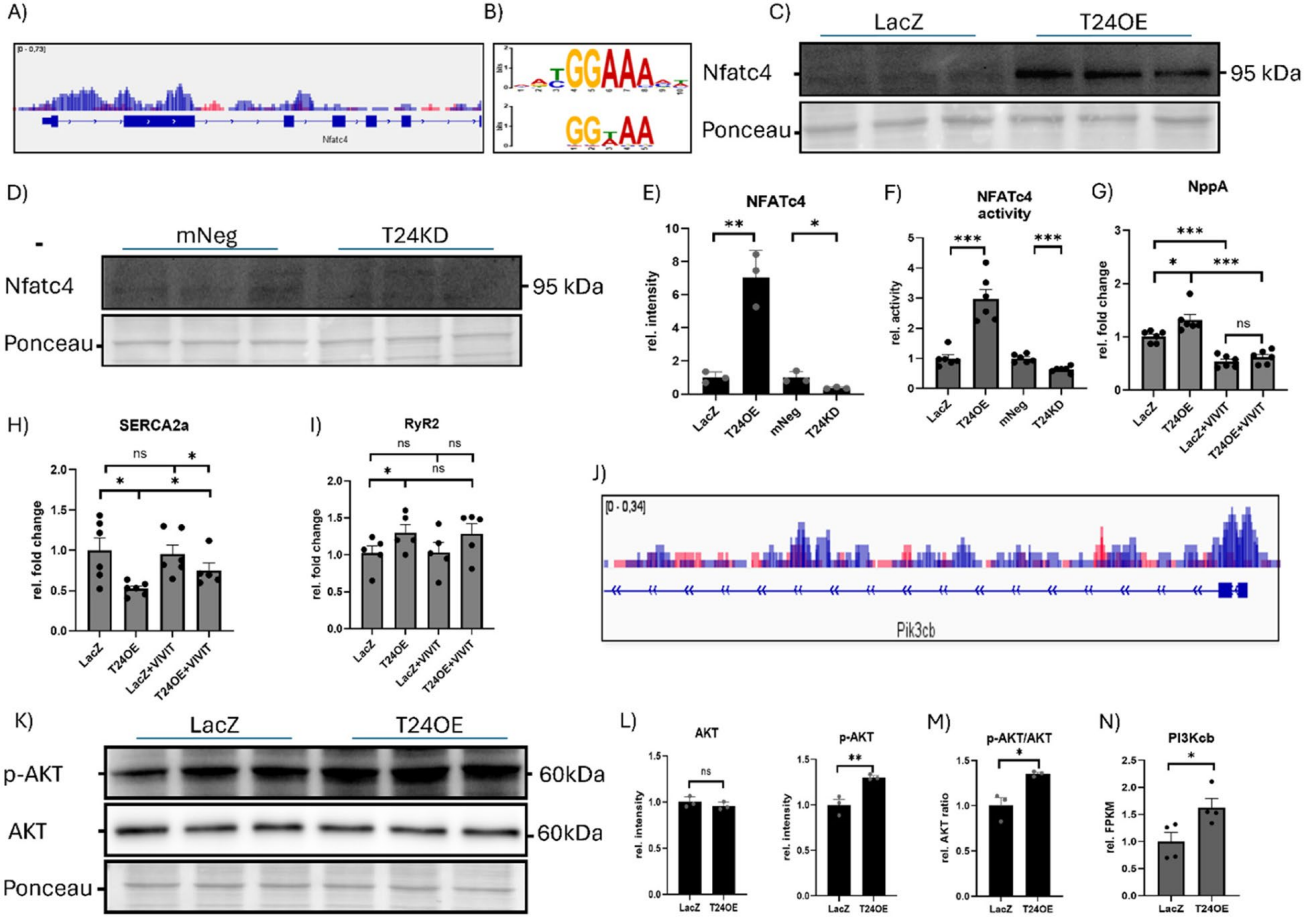
TRIM24 modulates calcium homeostasis and hypertrophy via NFATc4 and PI3K-AKT pathways. **A** TRIM24 enrichment peak obtained from the ChIP-seq data at the NFATc4 gene region. **B** NFAT-motif found in the TRIM24 ChIP-seq data motif analysis. Immunoblots and a bar graph showing densitometry analyses for NFATc4 in cardiomyocytes upon TRIM24 overexpression (T24OE, **C** & **E**) compared to LacZ control, and TRIM24 knockdown (T24KD, **D** & **E**). **F** Bar graph showing the effect of T24OE and T24KD on Luciferase activity determined using NFAT-reporter assay. Quantitative real-time PCR data using the RNA isolated from NRVCMs indicating the effect of TRIM24 overexpression on the levels of NppA (**G**), SERCA2a (**H**), and RyR2 (**I**), in the absence or the presence of NFAT inhibitor VIVIT. **J** TRIM24 enrichment peak obtained from the ChIP-seq data at the Pik3cb gene region. **K** Immunoblot for total AKT and its phosphorylated form (p-AKT) in protein isolates from the NRVCMs with LacZ and T24OE conditions. **L** Densitometry of AKT and p-AKT from immunoblot shown in J is presented as a bar graph. **M** Bar graph showing the ratio of densitometry of p-AKT and total AKT. **N** Transcript levels of Pik3cb from the RNA-seq data is presented as a bar graph. Statistical significance was determined using two-tailed Student's *t* test. *Error bars* show means ± SEM. *, *p* < 0.05; **, *p* < 0.01; ***, *p* < 0.001; *ns*, non-significant. Immunoblotting was conducted in three independent experiments with 3–6 biological replicates. Luciferase activity assay was performed in two or three independent experiments with 6 biological replicates per condition

To further uncover the mechanisms linking TRIM24 to calcium dysregulation and hypertrophic remodeling, we integrated RNA-seq, ChIP-seq, and proteomics data, identifying NFATc4 and the PI3K-AKT pathway as key mediators. ChIP-seq analysis revealed significant TRIM24 enrichment at the NFATc4 gene locus, with motif analysis further confirming NFATc4 binding motifs (Fig. 6A, B). Western blot analysis demonstrated that TRIM24 overexpression increased NFATc4 protein levels, while knockdown reduced it (Fig. 6C-E). This effect was functionally validated using a luciferase assay, which showed a significant increase in NFATc4 transcriptional activity upon TRIM24 overexpression as well as decreased activity upon knockdown (Fig. 6F). Given the potential role of NFATc4 in hypertrophy and SERCA2a repression, we investigated whether its upregulation contributed to the observed transcriptional changes. Treatment with the NFAT inhibitor VIVIT drastically reduced NppA expression, effectively abolishing the TRIM24-induced increase in this hypertrophic marker (Fig. 6G). Furthermore, while TRIM24 overexpression still reduced SERCA2a mRNA levels post-VIVIT treatment, the extent of downregulation was significantly mitigated, suggesting that NFATc4 is a mediator of TRIM24-driven SERCA2a repression (Fig. 6H). Conversely, RyR2 mRNA levels remained unaffected by NFAT inhibition, indicating an NFAT-independent regulatory mechanism (Fig. 6I).

Despite the downregulation of SERCA2a protein levels in TRIM24-overexpressing NRVCMs, contractility assays paradoxically demonstrated enhanced relaxation velocity (Fig. 5D). This discrepancy led us to explore compensatory mechanisms that might sustain SERCA2a activity despite reduced expression levels. Integrated analysis identified the PI3K-AKT pathway as a key downstream target of TRIM24, with ChIP-seq showing TRIM24 enrichment near genes encoding PI3K subunits and RNA-seq confirming a significant upregulation of PI3Kcb in TRIM24-overexpressing cells (Fig. 6J). Western blot analysis validated this finding, showing a substantial increase in phosphorylated AKT (p-AKT) levels, confirming TRIM24’s role in PI3K-AKT activation (Fig. 6K-N). The PI3K-AKT pathway enhances SERCA2a activity through phosphorylation of phospholamban (PLN), which relieves PLN's inhibitory effect on SERCA2a. Although no significant changes were observed in CamKII activation, Western blot analysis revealed a modest but significant increase in PLN phosphorylation at threonine sites, while serine phosphorylation showed an upward trend, albeit not statistically significant (Supplementary Fig. 7A-E). Consequently, the phosphorylated-to-total PLN ratio indicated a small shift towards increased phosphorylation, supporting a possible hypothesis that TRIM24 promotes SERCA2a activity via PI3K-AKT/PLN signaling (Supplementary Fig. 7F-G).

### TRIM24 alters calcium handling and induces hypertrophy in human ipsc-derived cardiomyocytes

**Fig. 7:**
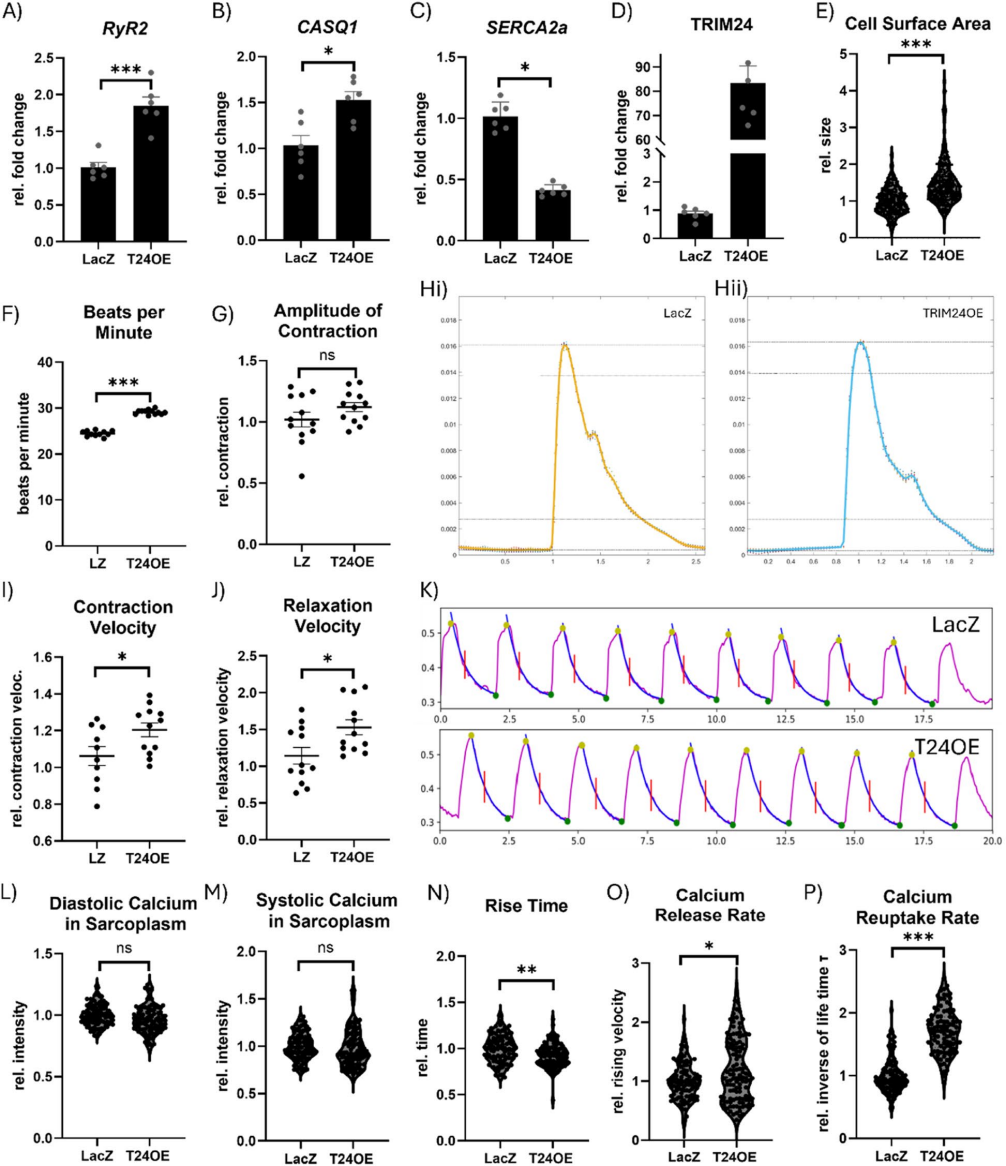
TRIM24 alters calcium handling and induces hypertrophy in human iPSC-derived cardiomyocytes. Quantitative real-time PCR results presented in the form of bar graphs indicating the altered levels of RyR2 (**A**), Casq1 (**B**), and SERCA2a (**C**) due to TRIM24 overexpression (T24OE) (**D**) in human induced pluripotent stem cell-derived cardiomyocytes (hiPSC-CMs). **E** Violin plot demonstrating the effect of T24OE on cardiomyocyte cell size in hiPSC-CMs. Cardiomyocyte contractility assay performed in hiPSC-CMs with T24OE compared to the LacZ control, indicates increased beating frequency (**F**), unaltered amplitude of contraction (**G**), and increased maximal contraction velocity (**I**) and relaxation velocity (**J**). **H** CCA measurement spectra for LacZ (Hi) and TRIM24 (Hii) indicating the overall contraction pattern changes. Calcium levels and flux measurement in iPSC-CMs (**K**) indicate the effect of TRIM24 overexpression on diastolic (**L**) and systolic (**M**) calcium levels, the time of calcium release (**N**), calcium release rate (**O**) and re-uptake (**P**) rates. qPCR was performed in two independent experiments with 6 biological replicates each. Cell surface area measurement was performed on two independent experiments in 3 biological replicates with at least 150 cells per condition. Contractility measurements were performed on two independent experiments with 6 biological replicates obtained from confluent cell layers imaged at 20 × magnification and averaged across all cells in the field of view which were then combined for the analysis. Calcium flux analyses were also based on two independent experiments, with 10 measurements per replicate, each consisting of 10 cells and 10 averaged contraction cycles, resulting in 100 averaged data points per condition. Statistical significance was determined using two-tailed Student's *t* test. *Error bars* show means ± SEM. *, *p* < 0.05; **, *p* < 0.01; ***, *p* < 0.001; *ns*, non-significant

To assess whether TRIM24 exerts comparable effects in human cardiomyocytes, we analyzed human induced pluripotent stem cell-derived cardiomyocytes (iPSC-CMs) following T24OE. A combination of qPCR, cell surface area analysis, contractility assays, and calcium imaging was employed. qPCR analysis revealed that TRIM24 overexpression in iPSC-CMs mirrored the transcriptional changes observed in neonatal rat ventricular cardiomyocytes (NRVCMs), with increased mRNA expression of *RyR2* and *Casq1*, and a reduction in *SERCA2a* mRNA levels (Fig. 7A–D). These results suggest that TRIM24 similarly modulates calcium handling gene expression in human cardiomyocytes. To determine whether TRIM24 also promotes a hypertrophic phenotype, we measured cell surface area. TRIM24 overexpression led to a significant increase in cell size in the magnitude of 1.5 to 2.5 times compared to controls (Fig. 7E; Supplementary Fig. 8), consistent with hypertrophic remodeling observed in NRVCMs.

Moreover, the results of the CCA revealed key similarities between NRVCMs and iPSC-CMs, including increased beating frequency and enhanced contraction and relaxation velocity in T24OE cells (Fig. 7F, I-J). However, unlike NRVCMs, iPSC-CMs did not exhibit a direct increase in contraction amplitude, suggesting a divergence in how TRIM24 influences contractile force across species (Fig. 7G). Contractility graphs further highlighted distinct deviations in the contraction cycle, with the second, smaller peak occurring later and at a lower amplitude, pointing to potential alterations in calcium handling rather than force generation (Fig. 7H).

Given this observation, we further measured calcium levels and flux in iPSC-CMs at a controlled pacing frequency of 0.5 Hz (Fig. 7K). Surprisingly, both diastolic and systolic sarcoplasmic calcium levels remained unchanged, which may explain the lack of increased contraction amplitude in T24OE iPSC-CMs (Fig. 7L, M). Despite stable overall calcium levels, the calcium transient curve exhibited significant alterations, with steeper rising and falling slopes resulting in reduced rise time (Fig. 7N). Whereas the increased calcium release rate corresponded with the higher contraction velocity observed in the CCA, confirming a TRIM24-mediated acceleration of excitation–contraction coupling (Fig. 7O). To evaluate calcium reuptake dynamics, an exponential decay function was fitted to the descending phase of the calcium transient, allowing for precise calculation of the calcium reuptake rate (Fig. 7K, blue curve). The results demonstrated a significantly accelerated reuptake in T24OE cells, consistent with the observed increase in relaxation velocity (Fig. 7P). These findings indicate that TRIM24 enhances calcium cycling efficiency, facilitating faster contraction and relaxation without directly increasing contractile force in iPSC-CMs. Overall, these results suggest that TRIM24 modulates cardiac function primarily by regulating calcium kinetics rather than maximal contractility in human iPSC-CMs.

## Discussion

The physiological role of TRIM24 in cancer and its association with transcriptional regulation is well established, with numerous studies in vitro, in animal models, and in humans highlighting its importance [3–6]. However, its role in cardiomyocytes remains largely unexplored. Our study provides the first comprehensive analysis of TRIM24's function in cardiac cells, revealing its critical role in chromatin remodeling, calcium homeostasis, and cardiomyocyte contractility. By integrating RNA-seq, ChIP-seq, proteomics, and functional assays, we have uncovered TRIM24 as a bidirectional transcriptional regulator with profound implications for cardiac physiology and pathology.

### TRIM24 as a bidirectional transcriptional regulator in cardiomyocytes

Our RNA-seq analysis identified TRIM24 as a bidirectional transcriptional regulator. The majority of suppressed pathways were related to the immune system, cytokine response, and mitochondrial metabolism, consistent with previous findings that TRIM24 suppresses interferon signaling [36, 37]. Conversely, upregulated pathways included lipid biosynthesis, actomyosin structure organization, and calcium regulation. Interestingly, cytoskeletal proteins such as Myl1 exhibited opposing trends at the mRNA and protein levels under TRIM24 manipulation, suggesting that TRIM24 may exert complex, multilayered regulatory control over cytoskeletal components. Specifically, the upregulation of Myl1 mRNA in TRIM24-overexpressing cells may be explained by the fact that MYL1 is a known transcriptional target of Serum Response Factor (SRF), a pathway that we have earlier found to be activated by TRIM24 through stabilization of the Dysbindin [24]. Conversely, the increased MYL1 protein levels observed in TRIM24 knockdown conditions may reflect post-transcriptional compensation, potentially involving altered mRNA translation or reduced protein degradation. These findings underscore the dual role of TRIM24 in modulating both transcriptional activity and protein stability, and are consistent with emerging evidence of other TRIM family members engaging in cytoskeletal remodeling through similarly multifaceted mechanisms [38]. Notably, the role of TRIM24 in lipid metabolism aligns with previous reports that its global loss leads to decreased expression of genes involved in steroid, fatty acid, and lipid metabolism [39]. However, our study is the first to link TRIM24 to cardiac contraction and calcium homeostasis, expanding its functional repertoire beyond cancer biology.

The ChIP-seq analysis on the other hand revealed a network of potential transcription factors interacting with TRIM24, shedding light on its multifaceted regulatory roles in cardiomyocytes. Known partners such as RaRα, previously linked to TRIM24, were confirmed, while novel interactions with eIF2, eIF4, ZNF416 and FOXO were identified. ZNF416, a pivotal regulator of fibroblast mechanoactivation, is co-occupied with H3K27ac, a preferred histone of TRIM24 [9, 40]. FOXO is associated with other members of the TRIM family [41] yet only FOXO4 is reported to interact with TRIM24 [43]. The FOXO family is known to be involved in diabetes-related cardiac metabolic abnormalities, potentially connecting this transcription factor to the altered mitochondrial and diabetes gene ontologies of our RNA-seq [44, 45]. eIF2 and eIF4 are involved in the circadian clock regulation [46], which is also known to be transcriptionally regulated by TRIM24 [46–48]. However, the proteomics data suggest that TRIM24 may exert opposing regulatory effects on the eIF2 and eIF4 pathways. This inverse pattern could indicate that TRIM24 modulates distinct translational programs depending on its expression level. Enhanced eIF2 signaling in TRIM24-overexpressing cells might reflect a shift toward selective translation typically associated with cellular stress or remodeling, while elevated eIF4 protein levels in the knockdown condition may point to increased global protein synthesis and growth-related activity. These observations may imply that TRIM24 fine-tunes the balance between selective and general translation in a context-dependent manner, potentially adapting protein synthesis to specific physiological demands.

Interestingly, the diverse ChIP-seq enrichment patterns observed for TRIM24 provide critical insights into its multifaceted role in chromatin regulation and transcriptional control. Sharp peaks near the transcription start site (TSS) suggest direct DNA binding at promoters or enhancers, while peaks farther from the TSS may reflect chromatin looping and 3D genome organization, highlighting TRIM24's role in spatial chromatin interactions. These peaks, influenced by interactions with transcription factors like RAR, can mediate either transcriptional activation or repression. Downstream peaks, particularly near genes like *Tfam*, may indicate RNA polymerase II termination or bidirectional promoter activity, linking TRIM24 to mitochondrial gene regulation. In contrast, broad enrichments spanning kilobases suggest widespread chromatin regulatory functions, often associated with repressive regions where the PHD and bromodomain of TRIM24 mediate histone reading. Notably, clusters of broad enrichment peaks, especially in H3K27ac-marked regions, point to active super-enhancers, while intense enrichments across gene bodies, such as at *Actb*, reflect RNA polymerase II pausing and elongation [49]. Evidence of TRIM24 ChIP-seq enrichment at established p53-binding loci, along with the identification of a p53 consensus DNA-binding motif, suggests a potential regulatory interaction between TRIM24 and p53 signaling in NRVCMs. This observation aligns with previous findings in other cell types, where TRIM24 has been shown to promote p53 ubiquitination, thereby contributing to chromatin remodeling and transcriptional repression [8, 23]. These diverse patterns underscore the ability of TRIM24 to modulate chromatin states through its E3 ligase activity, histone reader function, and co-transcriptional regulation via the LxxLL motif in cultured cardiomyocytes. Collectively, these findings suggest TRIM24 as a central player in nuclear proteostasis, orchestrating transcriptional homeostasis and chromatin architecture in cardiomyocytes.

### The impact of TRIM24 on chromatin structure and transcriptional regulation

To further understand how TRIM24 influences transcriptional regulation, we analyzed changes in chromatin organization using super-resolution microscopy. TRIM24 knockdown led to elevated H3K4me3 levels and increased chromatin openness, indicative of a shift toward a transcriptionally active euchromatin state. Conversely, TRIM24 overexpression reduced H3K4me3 levels and chromatin cluster organization, suggesting a shift towards a more repressive chromatin state, aligning with the predominantly downregulated gene expression observed in cardiomyocytes upon TRIM24 overexpression. These findings suggest that TRIM24 restricts chromatin accessibility, potentially through its interaction with p53 and histone methylation patterns, as previously reported [23]. Intriguingly, the residual H3K4me3 signal in TRIM24-overexpressing cells was highly locus-specific, implying that transcriptional activity is confined to specific genomic regions, such as those involved in lipid biosynthesis or cytoskeletal organization. This localized regulation was further supported by our analysis of H3K27ac, a known TRIM24 binding partner [50]. TRIM24 overexpression increased H3K27ac cluster density without altering cluster size, indicating a highly targeted transcriptional activation effect. These results underscore TRIM24's dual role as a transcriptional regulator, with its locus-specific activity potentially explaining the sharp ChIP-seq enrichment peaks observed. Collectively, these findings highlight TRIM24's critical role in shaping the chromatin landscape, maintaining a delicate balance between transcriptional repression and activation in cardiomyocytes.

### The role of TRIM24 in calcium homeostasis and cardiomyocyte contractility

Our integrated RNA-seq, ChIP-seq, and proteomics analyses revealed a potential role of TRIM24 in cardiomyocyte calcium homeostasis, prompting a detailed investigation of key calcium-handling proteins: CASQ1, RyR2, and SERCA2a. TRIM24 overexpression upregulated CASQ1 and RyR2 while downregulating SERCA2a, whereas TRIM24 knockdown reduced CASQ1 and RyR2 levels without significantly altering SERCA2a. This pattern is consistent with trends observed in the RNA-seq data, where CASQ1 and RyR2 showed increased expression in the TRIM24 overexpression condition. At the proteomic level, CASQ1 was significantly downregulated in TRIM24 knockdown samples, further supporting its regulation by TRIM24. RyR2 and SERCA2a were not detected in the proteomic dataset, likely due to their membrane-associated nature and known detection limitations in mass spectrometry workflows. Intriguingly, qPCR analysis showed increased RyR2 mRNA in TRIM24 knockdown cells, a discrepancy likely attributable to the cytotoxic effects of TRIM24 depletion, which may impair protein synthesis despite elevated mRNA levels [23]. This could also explain the failure of TRIM24 knockdown NRVCMs to exhibit measurable contractility in cardiomyocyte contractility assays. To further explore the impact of TRIM24 on calcium homeostasis, we employed immunoblotting and super-resolution microscopy to assess the structural organization of calcium channels. TRIM24 overexpression significantly increased RyR2 expression, leading to larger and more RyR2 clusters. This aberrant clustering, deviating from control conditions and prior reports [51–53], likely underlies the observed increase in maximal contraction velocity and calcium flux. The overabundance of Casq1, driven by TRIM24 binding to the CASQ1 gene, may further exacerbate this dysregulation. While CASQ2 is the primary cardiac isoform, the altered inhibitory effect of CASQ1 on RyR2 under low sarcoplasmic reticulum Ca^2^⁺ conditions could disrupt calcium-induced calcium release, leading to rapid calcium release and arrhythmogenic potential [54, 55]. Additionally, the beta-adrenergic signaling pathway, enriched in our KEGG analysis, implicates protein kinase A and CaMKII in RyR2 phosphorylation, potentially increasing its opening probability and contributing to the observed calcium dysregulation.

In contrast, SERCA2a exhibited a loss of structural cluster organization in TRIM24-overexpressing cells, with persistent homology analysis revealing less dense clusters at the periphery. This disorganization, typical of hypertrophic conditions, may impair the ability of SERCA2a to transport Ca^2^⁺ efficiently [56]. However, the increased relaxation velocity and calcium reuptake observed in our CCA and calcium flux assays suggest compensatory mechanisms. The overabundance of CASQ1, with its higher calcium-binding capacity, may reduce the electrical gradient against which SERCA2a pumps, enhancing its efficiency. Furthermore, enrichment at PI3K subunits and upregulation of *Pi3kcb* mRNA suggests activation of the PI3K-AKT pathway upon TRIM24 overexpression. Although the observed effects were modest, we believe that the upregulated pathway is likely to inhibit phospholamban and enhance SERCA2a activity, consistent with previously reported findings in the literature [57, 58]. These findings highlight the dual role of TRIM24 in calcium handling, balancing the dysregulation of RyR2 and SERCA2a through both direct and indirect mechanisms.

To assess the relevance of these findings in human cells, we conducted CCA in iPSC-derived cardiomyocytes (iPSC-CMs). Like NRVCMs, TRIM24 overexpression increased beating frequency and contraction/relaxation velocity, but contraction amplitude was only enhanced in NRVCMs. This discrepancy may stem from fundamental species-specific and age-specific differences in calcium handling, as iPSC-CMs maintain stable systolic and diastolic calcium levels, whereas NRVCMs, lacking a transverse-axial tubule system, depend on subsarcolemmal calcium influx for contraction [59]. Nonetheless, the altered contraction dynamics in iPSC-CMs confirm that TRIM24’s role in calcium homeostasis is conserved, albeit with species-specific nuances. Collectively, these findings establish TRIM24 as a key regulator of calcium homeostasis, balancing RyR2 and SERCA2a dysregulation through direct transcriptional control and secondary compensatory mechanisms. While its contractility-enhancing effects are more pronounced in NRVCMs, TRIM24 still modulates calcium flux and contraction dynamics in human iPSC-CMs, emphasizing its potential relevance in cardiac pathophysiology across species.

**Fig. 8:**
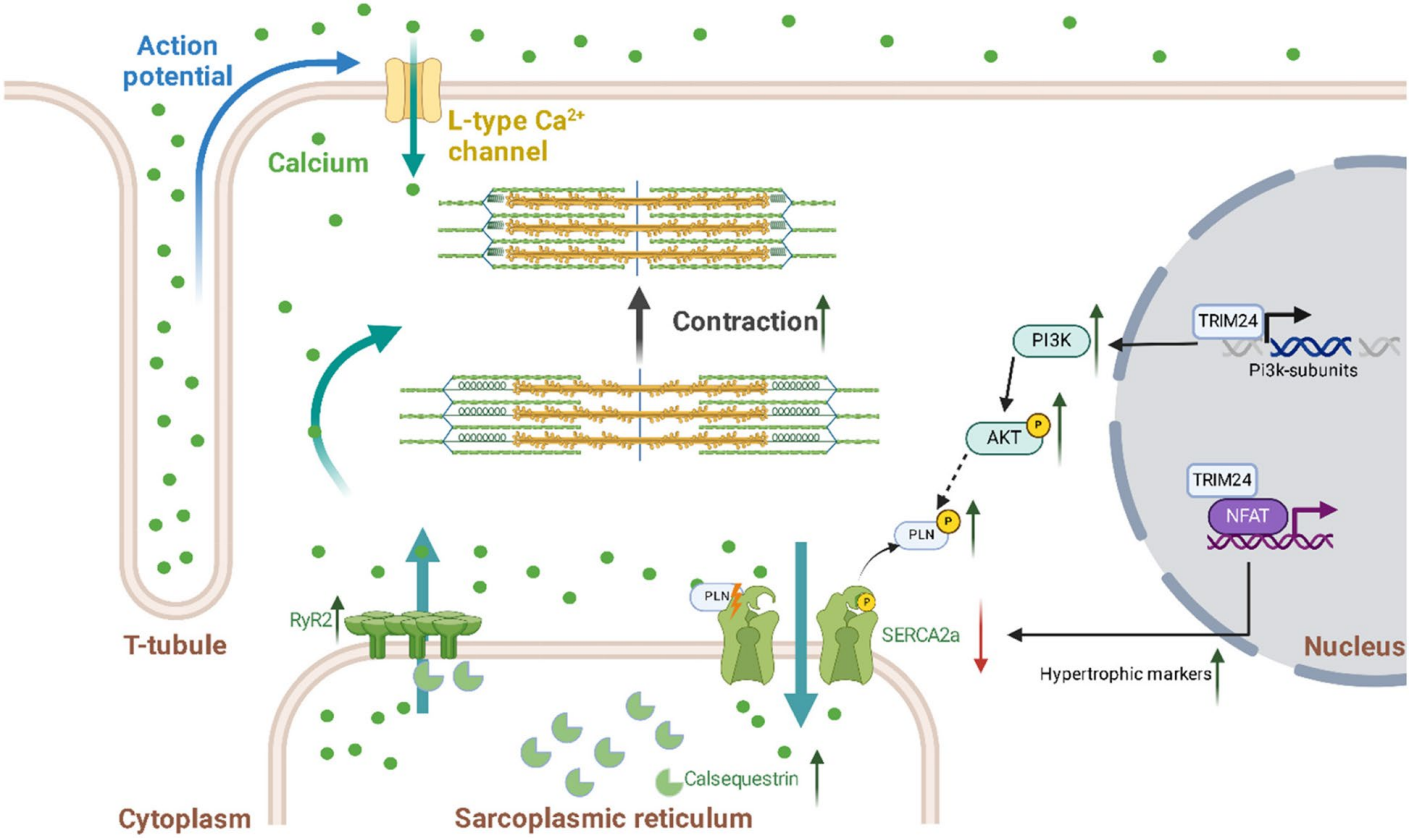
TRIM24 as a key regulator of chromatin remodeling, transcriptional control, and calcium homeostasis in cardiomyocytes. This schematic illustrates the multifaceted role of TRIM24 in cardiomyocyte function, integrating its effects on chromatin dynamics, transcriptional regulation, calcium signaling, and contractility via modulating the levels, organization, and/or activities of Calsequestrin, Ryanodine receptor 2 (RyR2), SERCA2a, Phospholamban, and PI3K/NFAT signaling

In summary, our study establishes TRIM24 as a pivotal regulator of cardiomyocyte function, orchestrating chromatin dynamics, transcriptional regulation, and calcium homeostasis in a highly context-dependent manner (Fig. 8). By integrating multi-omics approaches, we reveal TRIM24 as a bidirectional transcriptional regulator that fine-tunes immune response, lipid metabolism, and contractile machinery while modulating chromatin accessibility through histone interactions. Furthermore, our findings uncover a direct role for TRIM24 in calcium signaling, influencing RyR2 and SERCA2a function. The interplay between TRIM24-mediated chromatin remodeling and transcriptional control underscores its broader significance in cardiac physiology, beyond its well-established role in cancer biology. These insights not only expand our understanding of the multifaceted functions of TRIM24 but also suggest its potential as a therapeutic target for cardiac dysfunction, warranting further investigation into its mechanistic roles in human cardiomyopathies.

## Supplementary Information

Supplementary Material 1.

Supplementary Material 2.

Supplementary Material 3.

## Data Availability

All data underpinning the findings of this study are comprehensively provided within the manuscript or in supplementary data file. The mass spectrometry proteomics data have been deposited to the ProteomeXchange Consortium via the PRIDE 59 partner repository with the dataset identifiers PXD062686 and PXD064832.
