# Correction: TRIM24 regulates chromatin remodeling and calcium dynamics in cardiomyocytes

**DOI:** 10.1186/s12964-025-02352-3

**Published:** 2025-07-15

**Authors:** Marco Neu, Anushka Deshpande, Ankush Borlepawar, Elke Hammer, Ahmed Alameldeen, Phillipp Vöcking, Timon Seeger, Michael Hausmann, Norbert Frey, Ashraf Yusuf Rangrez

**Affiliations:** 1 Department of Internal Medicine III (Cardiology and Angiology), University Hospital Heidelberg, Im Neuenheimer Feld 410, 69120 Heidelberg, Germany; 2 German Center of Cardiovascular Research (DZHK), Partnersite Heidelberg/Mannheim, Heidelberg, Germany; 3 Kirchoff‑Institute for Physics, University of Heidelberg, Im Neuenheimer Feld 227, 69120 Heidelberg, Germany; 4 Cellular Adaptation and Bioenergetics Group, Institute for Translational Medicine, Medical School Hamburg, Am Kaiserkai 1, 20457 Hamburg, Germany; 5 Interfaculty Institute of Genetics and Functional Genomics, University of Greifswald, Felix‑Hausdorff‑Straße 8, 17475 Greifswald, Germany; 6 German Center of Cardiovascular Research (DZHK), Partnersite Greifswald, Greifswald, Germany

**Correction**: ***Cell Commun Signal 23, 312 (2025)***

10.1186/s12964-025-02323-8

In this article, the author name Ahmed Alameldeen appeared incorrectly as Ahmed Ahlmaldeen.

The author group has been updated above and the original article has been corrected.

