# Supplementary material for: TRIM24 regulates chromatin remodeling and calcium dynamics in cardiomyocytes
Source: Cell Commun Signal. 2025 Jul 1;23:312. doi: 10.1186/s12964-025-02323-8 (PMC12211185; doi:10.1186/s12964-025-02323-8)
Supplement: Supplementary file 2 — Supplementary Material 2. [file 12964_2025_2323_MOESM2_ESM.docx]

**TRIM24 orchestrates chromatin remodeling and calcium dynamics in cardiomyocytes**

**Marco Neu^1,2,3^, Anushka Deshpande^1,2^, Ankush Borlepawar**^4^**, Elke Hammer^5,^**^6^**, Ahmed Ahlmadeen^1,2^, Phillipp Vöcking^1,2^, Timon Seeger^1,2^, Michael Hausmann^3^, Norbert Frey^1,2^,** **and Ashraf Yusuf Rangrez^1^**^,2,^*

^1^ Department of Internal Medicine III (Cardiology and Angiology) University Hospital Heidelberg, Im Neuenheimer Feld 410, 69120 Heidelberg, Germany

^2^ German Center of Cardiovascular Research (DZHK), Partnersite Heidelberg/Mannheim, Heidelberg, Germany

^3^ Kirchoff-Institute for Physics, University of Heidelberg, Im Neuenheimer Feld 227, 69120 Heidelberg, Germany

^4^ Cellular Adaptation and Bioenergetics Group, Institute for Translational Medicine, Medical School Hamburg, Am Kaiserkai 1, 20457 Hamburg, Germany

^5^ Interfakultäres Institut für Genetik und Funktionelle Genomforschung, University of Greifswald, Felix-Hausdorff-Straße 8, 17475 Greifswald, Germany

^6^ German Center of Cardiovascular Research (DZHK), Partnersite Greifswald, Greifswald, Germany

*Correspondence: ashrafyusuf.rangrez@med.uni-heidelberg.de

**Supplementary Table 1: Oligonucleotide sequences of primers used for the quantitative real-time PCR. hs – Homo sapiens; rn – Rattus norvegicus**

| Primer name | Sequence | Gene |
| --- | --- | --- |
| hs_TRIM24_ rt-fw | 5'-AGCCTAGCTCAATTACGGCTC-3' | TRIM24 |
| hs_TRIM24_ rt-rev | 5'-GCGGTTGCTGATGAGAGATGG-3' |  |
| rn_ CASQ1_ rt-fw | 5'-GACAAGGGTGTTGGCTTTGG-3' | CASQ1 |
| rn_ CASQ1_ rt-rev | 5'-GGGTCTTCTAGGACATCGAGC-3' |  |
| rn_ NppA_ rt_ fw | 5'-GGAGCAAATCACAGTG-3' | NppA |
| rn_ NppA_ rt_ rev | 5'-ACCTCATCTTCGGCAT-3' |  |
| rn_ Serca2a_ rt_ fw | 5'-GGGTGCGTGCATGTGCGTTG-3' | SERCA2 |
| rn_ Serca2a_ rt_ rev | 5'-ACACAGTGAGCTGGGGGCTGT-3' |  |
| rn_ RPL32_ rt_ fw | 5'-GGTGGCTGCCTTTACG-3' | RPL32 |
| rn_ RPL32_ rt_ rev | 5'-CCGCACCCTGCAATGC-3' |  |
| rn_ RyR2_ rt_ fw | 5'-ATCCCAACGCAGCAAGGAAA-3' | RyR2 |
| rn_ RyR2_ rt_ rev | 5'-TTCACCTTTGCTGGCACTGA-3' |  |

**Supplementary Table 2: Enriched DNA motifs and associated transcription factors identified in TRIM24 ChIP-seq**

| **Possible TF** | **p-value** |
| --- | --- |
| RaRα | 4.51e-03 |
| TP53 | 1.54e-03 |
| NFATc4 | 3.70e-03 |
| YY2 | 1.33e-04 |
| STAT1/STAT3 | 1.47e-04 |
| NHLH2 | 9.62e-04 |
| FOXO1 | 7.95e-04 |
| FOXO3 | 5.75e-04 |
| FOXO4 | 6.71e-04 |
| TFDP1 | 7.64e-05 |
| FOXI1/FOXL/FOXg1 | 4.02e-03 |
| ZNF416 | 3.98e-03 |
| ZNF75a | 8.35e-04 |
| ELF2/ELF4 | 2.39e-03 |
| SPDEF | 1.50e-04 |
| RBPJ | 6.55e-05 |
| ZFP57 | 3.06e-03 |
| ZBTB12 | 6.35e-05 |
| SIX2 | 3.44e-03 |
| ZNF93 | 9.65e-04 |
| TCF4 | 7.21e-04 |
| MYC | 4.10e-03 |
| ZIC1/ZIC2 | 5.37e-04 |

Supplementary text: Persistent homology analysis

A major principle to characterize the meaning of “topology” or “topological analysis” is to record properties of structures (depicted in a pointillist manner) which are invariant under certain deformations of the object. Mathematically these deformations correspond to continuous transformations of the topological space defined by the structures. Deformations which might fragment the structures are excluded. In the following, the attention will be focused on two quantifiable properties: (a) the number of components which are independent from each other in such sense that connections between points (which represent fluorophore blinking events during the localization microscopy measurement and therefore the labeled proteins) only exist within the respective components; (b) the number of holes of the structures inside the components. In algebraic topology, these properties are called the Betti numbers for zero dimensional and one dimensional simplicial complexes, respectively. They turn out to be very important topological invariants which help to distinguish between different topological spaces.

By comparing these quantities to two objects, it can be decided whether they have the same topology or not. Localization microscopy images are actually point-sets defined by the location of the fluorophores. Thus, an appropriate method is required by which components and holes can be defined. To accomplish this, the point-set is converted into an object as described by the following procedure: First, the point-set is defined by the coordinates of blinking labeling points. In the next step, a geometric relationship among the points is defined by growing spheres of radius α around each of them. Whenever two spheres mutually embed in each-other’s center, these centers of the growing spheres are connected by an edge. Points connected in that way are considered to belong to the same component. Any two points which are connected by a path through the existing edges are in the same component. Increasing the radii of the spheres, further points are reached connecting two previously disjoint components. Thus, one can follow how the number of components is changing as a function of the increasing radius α. This means that each point is a separate component at the beginning, whereas for an increasing radius being large enough, each point is connected with each other. At the end of the procedure a single component is remaining.

The definition of holes also stems from this process. In order to build a solid, beside points and lines, face building blocks are required. For this, the simplest polygon, the triangle is appropriate. Whenever three edges form a triangle, not only the edges but the face of the triangle is considered. The described procedure is presented in [Figure 3](https://www.mdpi.com/1422-0067/19/8/2263#fig_body_display_ijms-19-02263-f003)D for a particular γH2AX cluster taken from Hoffmann et al. Once the surfaces are defined, the holes are counted. In fact, it is possible to register their number and the number of components for every separate value of the radius α.

From single-molecule localization to topological abstraction via barcode representation: A single-molecule localization microscopy (SMLM) density map of a SkBr3 cell nucleus 30 minutes after exposure to 2 Gy irradiation shows heterochromatin labeled by H3K9me3 (green) and γH2AX-marked DNA damage foci (red). A zoomed-in view highlights a representative γH2AX cluster, which is shown as a scatter plot where each point corresponds to an individual fluorophore localization. Topological reconstruction of this cluster is achieved using α-shape filtration at increasing radius values (α = 30, 45, 90, 180 nm). In this process, edges are drawn between points when their surrounding spheres mutually contain each other’s centers, and triangular connections are filled in as surface elements to build up the complex geometry of the structure. The resulting topological features are summarized in barcode plots, where dimension-0 barcodes represent connected components and dimension-1 barcodes capture topological holes, each persisting across defined α-scales.


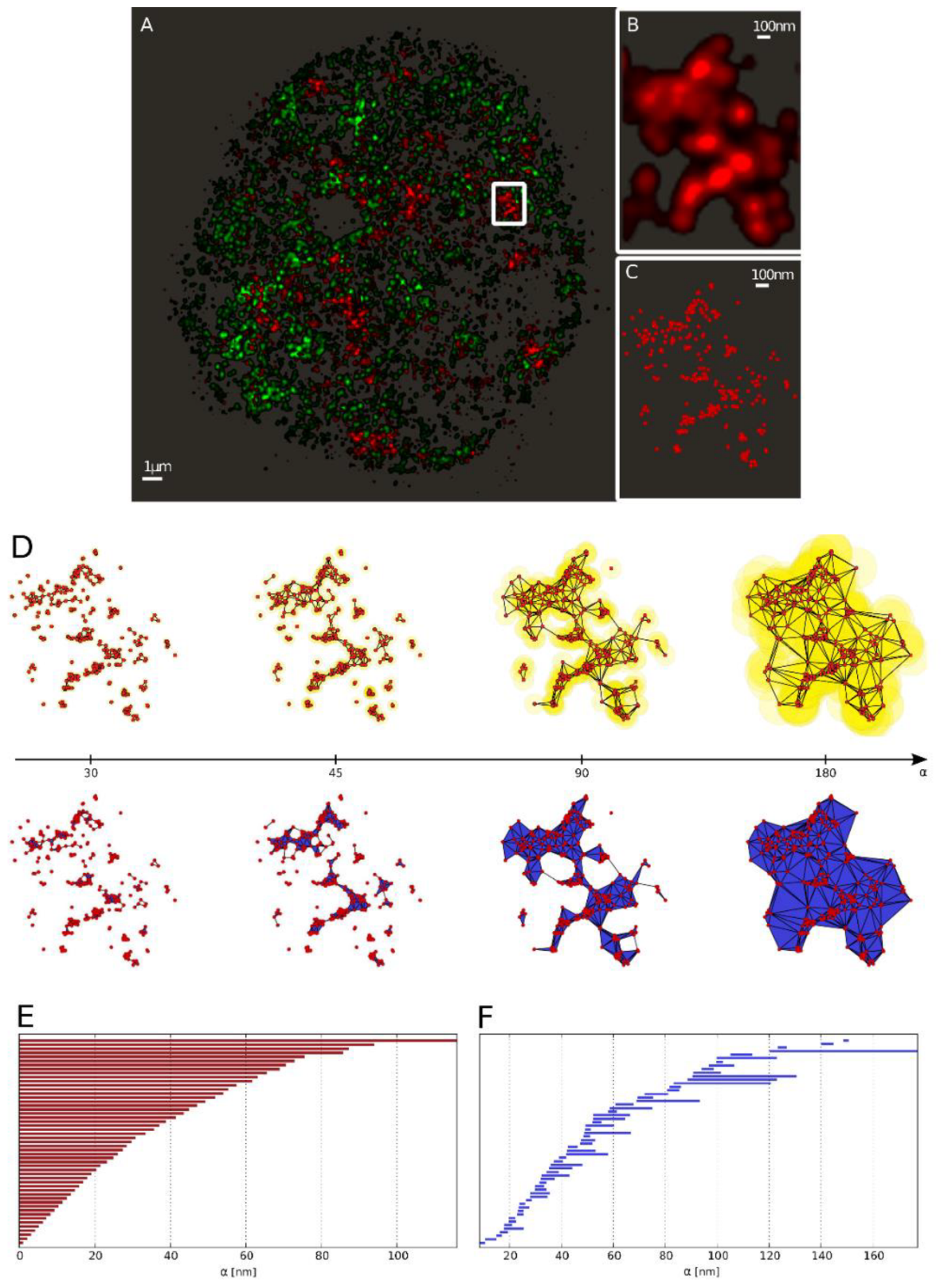


Supplementary Figure 1: **Persistent Homology analysis visualization.** (A) SMLM-based density image showing a full view of the nucleus of a SkBr3 cell 30 minutes post-irradiation with 2 Gy, visualizing heterochromatin (green; H3K9me3-labeled) and γH2AX-marked DNA damage sites (red). (B) Magnified region highlighting a selected γH2AX focus. (C) Corresponding scatter plot of this region, where each dot represents an individual fluorophore localization. (D) Topological reconstruction of the γH2AX cluster using α-shape filtration for increasing radius values α = {30, 45, 90, 180} nm. In the upper panels, connections (edges) are drawn between fluorophore centers when their associated spheres overlap mutually. In the lower panels, triangles formed by three edges are filled as surface elements, progressively shaping the topological space. (E) Barcode plot representing dimension-0 features (connected components) as a function of α. (F) Barcode plot for dimension-1 features (topological holes), capturing their persistence across scales. The graphs shown in this study reflect a smoothed envelope of the histogram of barcode endpoints, summarizing the persistence distribution of topological features.

Image adapted from:

Hofmann, Andreas, et al. "Using persistent homology as a new approach for super-resolution localization microscopy data analysis and classification of γH2AX foci/clusters." *International journal of molecular sciences* 19.8 (2018): 2263.


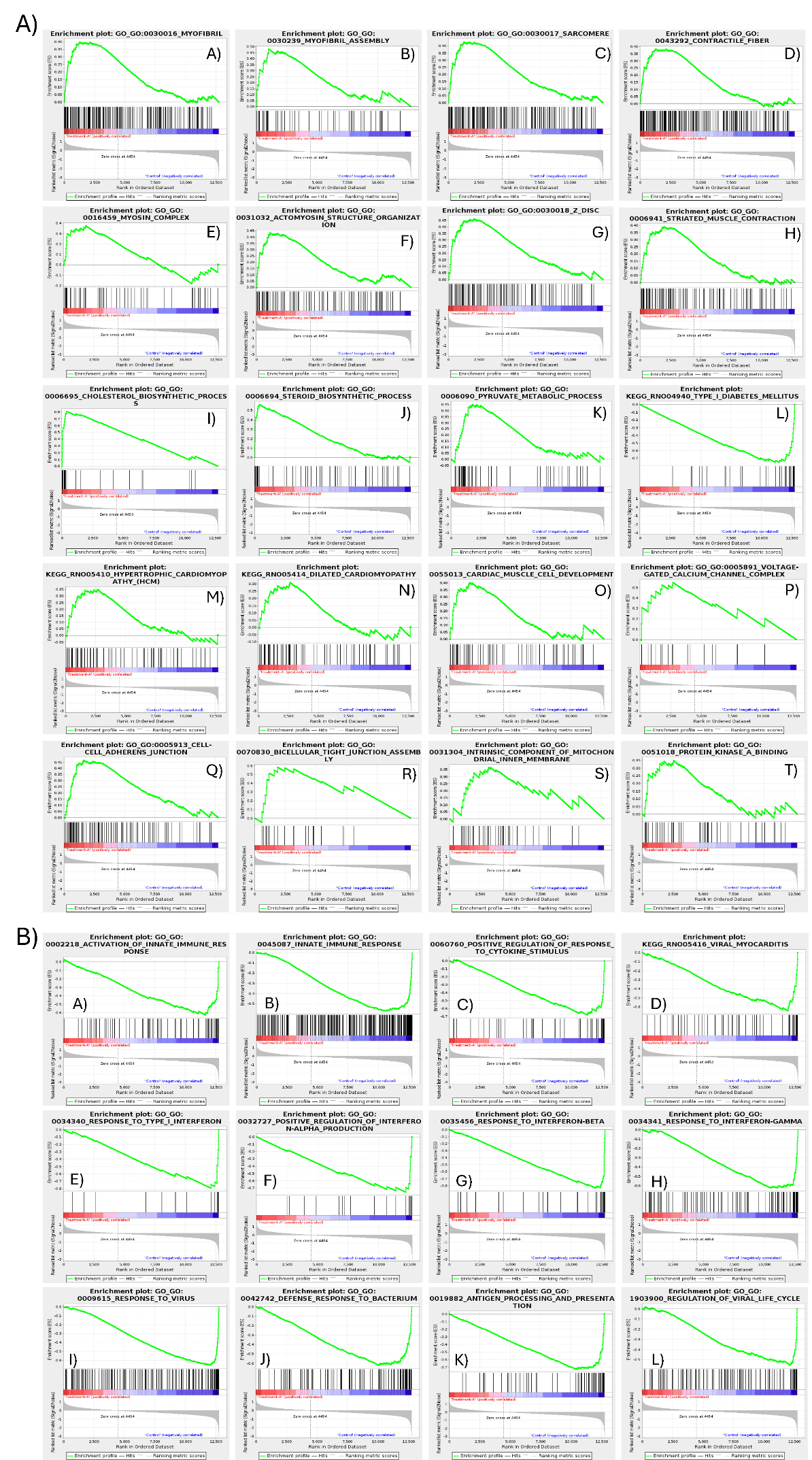


**Supplementary Figure 2:** **TRIM24 modulates cardiomyocyte transcriptome. A**) GSEA-plots indicating the effects of TRIM24 overexpression on lipid metabolsim, myopathies, cytoskeleton, calcium homeostasis and cell-cell connections**. B**) GSEA-plots indicating the inhibitory effects of TRIM24 overexpression on lipid metabolsim, myopathies, cytoskeleton, calcium homeostasis and cell-cell connections.


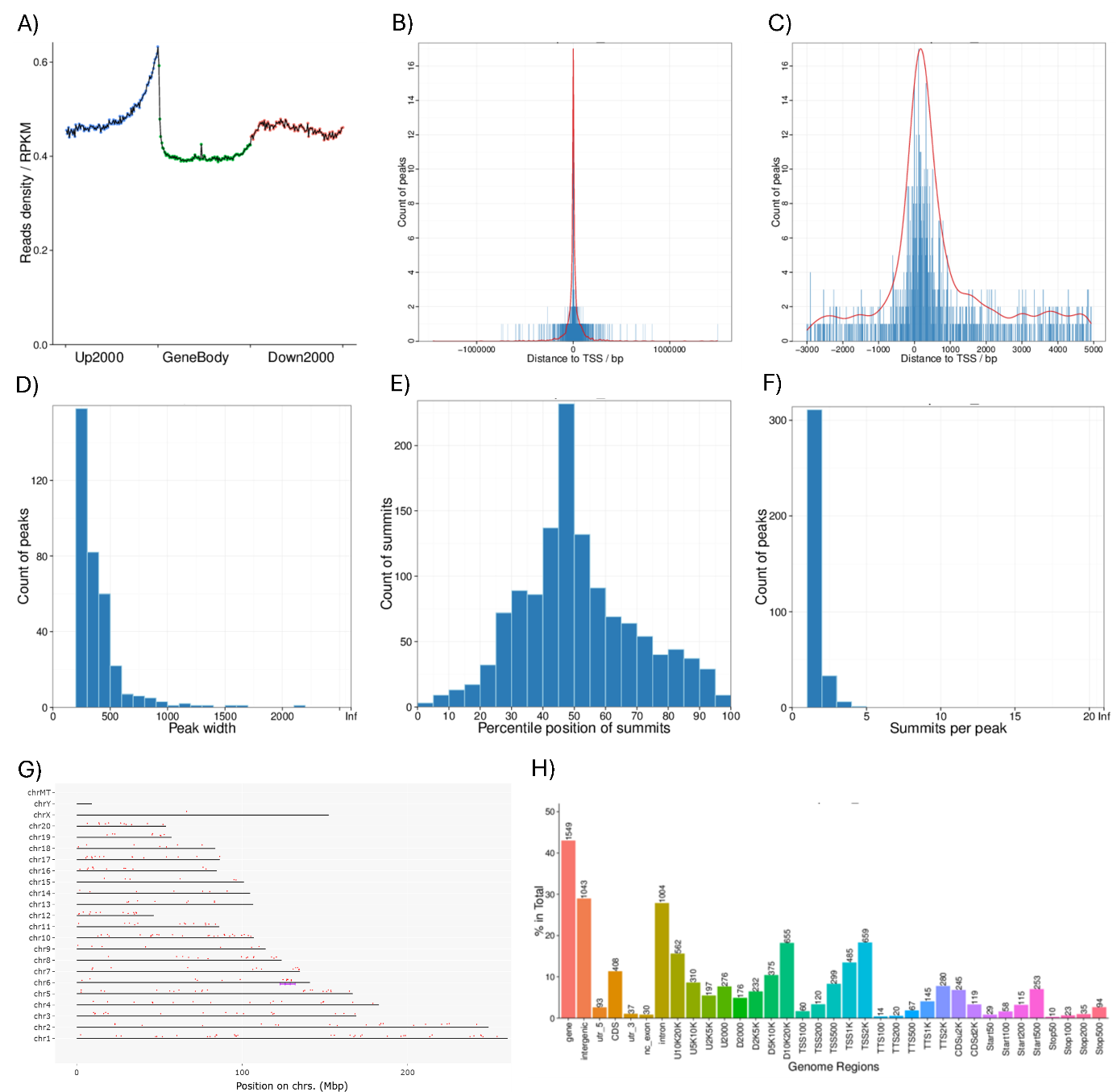


**Supplementary Figure 3:** **TRIM24 interacts with transcription factors and modulates cardiac gene regulation**. **A**) Distribution of the reads mapped to the gene. Since transcription factor and histone binding sites are key to gene regulation, analyzing their positional distribution helps infer protein function. Each gene, including 2 kb upstream and downstream, is divided into 100 segments, and read density is calculated as the mapped read count relative to the total reads. **B**) Peak-TSS distance distribution. blue: Reads histogram, red: Kernel Density Estimate. The enrichment density is high near the TSS. **C**) Enlarged image of the peak shown in B, where the highest enrichment density can be found in the genomic region closely behind the TSS. **D**) Example of the Distribution of peak width in one replicate. The peak width here represents the length of the DNA that is bound by TRIM24. **E**) Example of the Summits distribution in one replicate. Each peak is divided to 100bp windows and the summits in each window of all the peaks are counted. **F**) Example of Count of summits in peaks in one replicate. The number of summits in each peak is analysized, which infers the peak type in the IP experiment. **G**) Enrichment location distribution. **H**) Histogram of TRIM24 binding across genomic regions: TRIM24 binds not only to gene and intergenic regions but also to various other genomic loci.


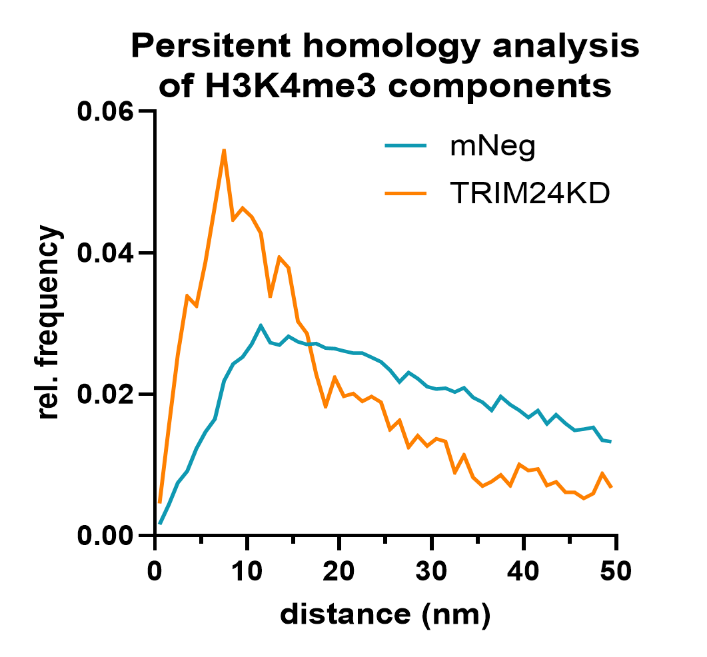


**Supplementary Figure 4:** **Level of TRIM24 significantly affects the state of chromatin landscape. A**) Persistent homology analysis of H3K4me3 components in NRVCMs upon TRIM24 knockdown (TRIM24KD) compared to the negative control (mNeg).


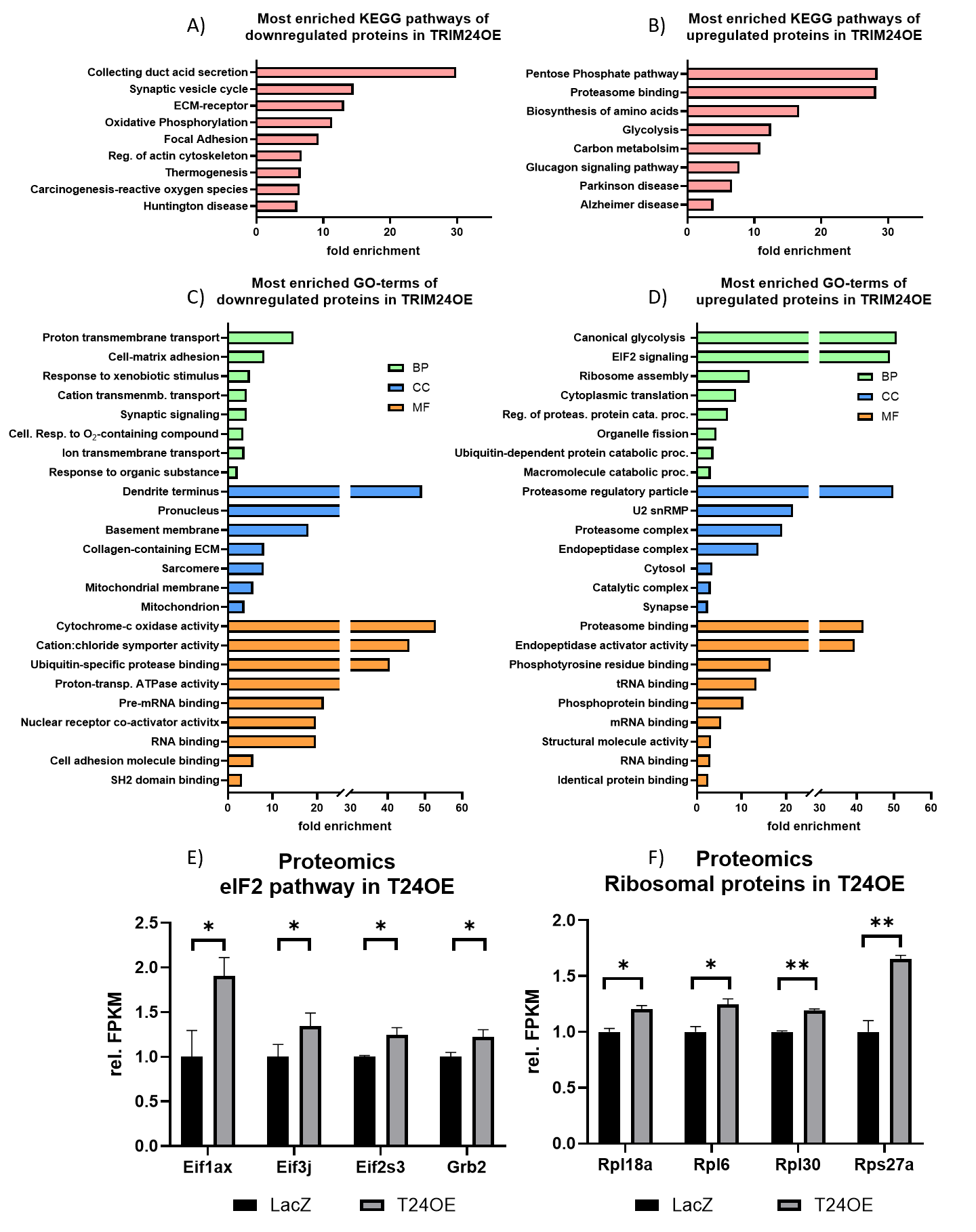


**Supplementary Figure 5:** **Proteomics analysis of TRIM24 overexpressing NRVCMs** **A**) Most enriched KEGG pathways of downregulated proteins in TRIM24OE. **B)** Most enriched KEGG pathways of upregulated proteins in TRIM24OE **C)** Most enriched GO-terms of downregulated proteins in TRIM24OE. **D)** Most enriched GO-terms of upregulated proteins in TRIM24OE. **E)** TRIM24 overexpression causes an elevated eIF2 pathway. **F)** Ribosomal proteins are elevated in TRIM24 overexpressing NRVCMs.


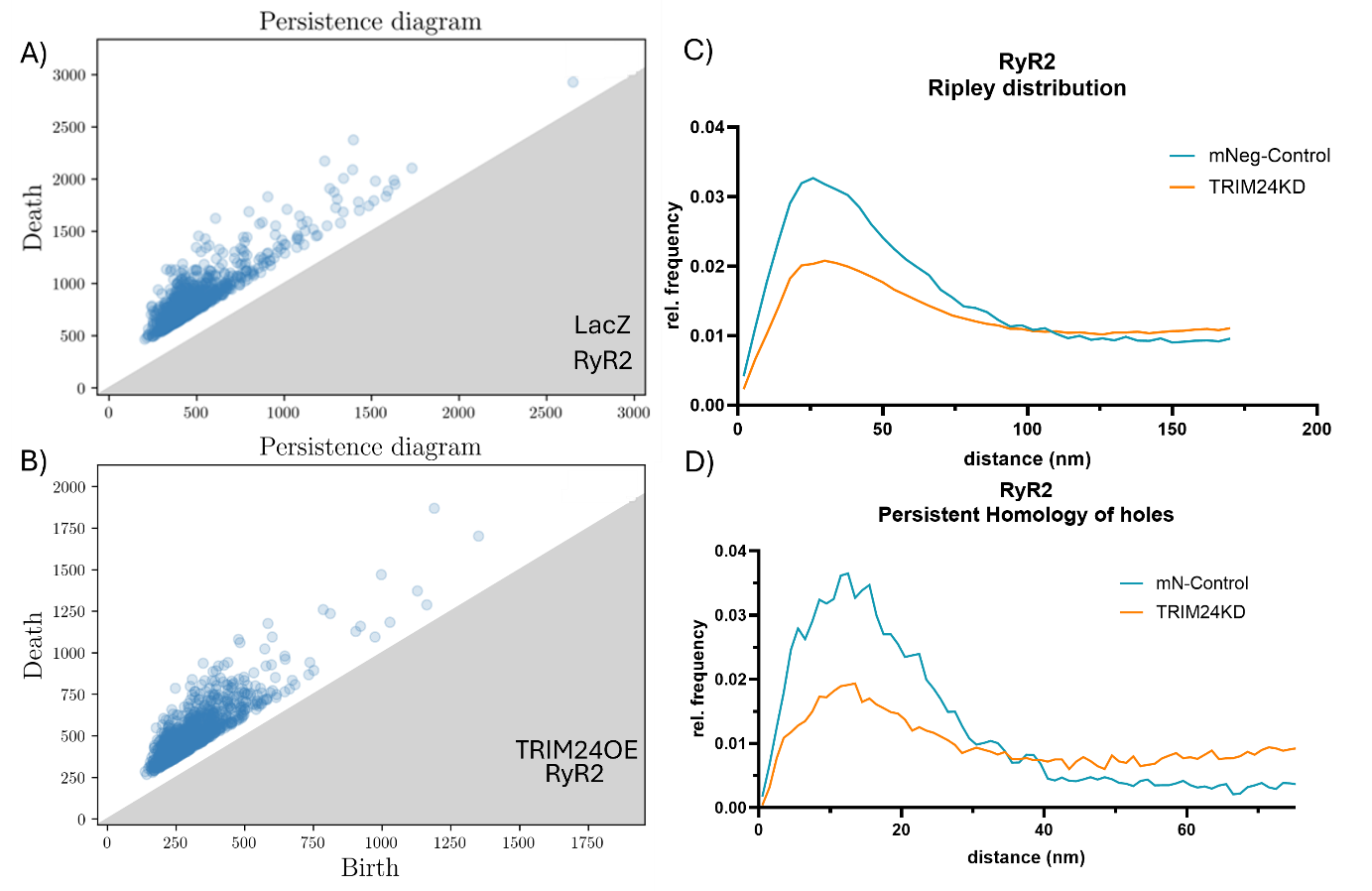


**Supplementary Figure 6: TRIM24 alters cardiomyocyte contractility by regulating the levels and organization of CASQ1, RyR2, and SERCA2a.** **A+B**) Persistence diagram of RyR2 holes in LacZ-Control and TRIM24 overexpressing NRVCMs. RyR2 clusters in TRIM24OE NRVCMs are denser. **C**) Ripley analysis of RyR2 in TRIM24 knockdown NRVCMs. Absence of TRIM24 leads to a loss of RyR2 organisation structure. **D**) Persistent homology analysis of topological RyR2 holes in TRIM24 knockdown NRVCMs. Statistical significance was determined using two-tailed Student's t test. Error bars show means ± S.E. *, p < 0.05; **, p < 0.01; ***, p < 0.001; ns, non-significant.

**
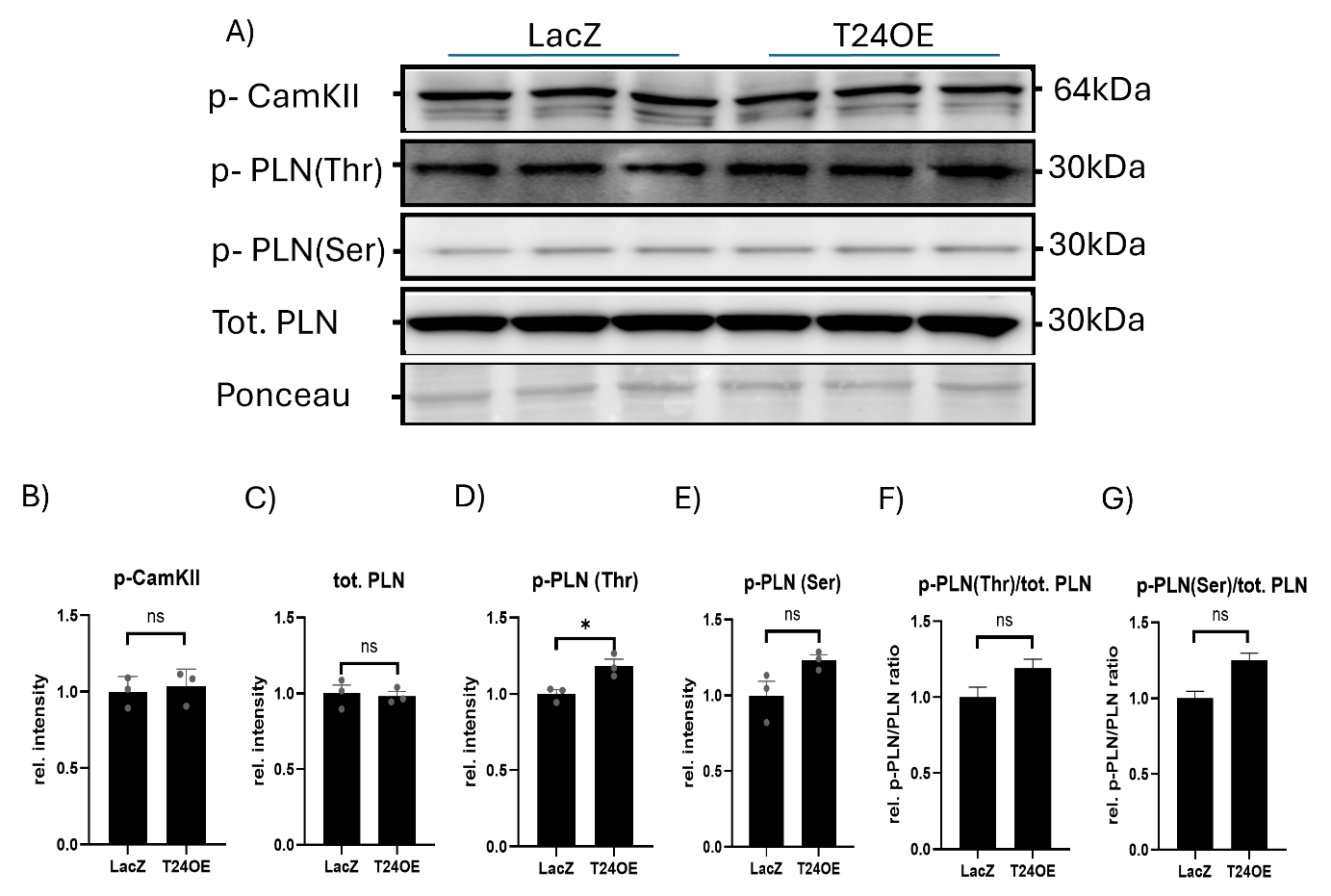
Supplementary Figure 7:**

**A**) Immunoblot image for p-CamKII as well as total, serin-phosphorylated and threonine-phosphorylated phospholamban (PLN). Respective densitometry analyses are depicted in **B-E**. Ratio of threonine-phosphorylated (**F**) or serine-phosphorylated (**G**) to total PLN is depicted as bar graphs.


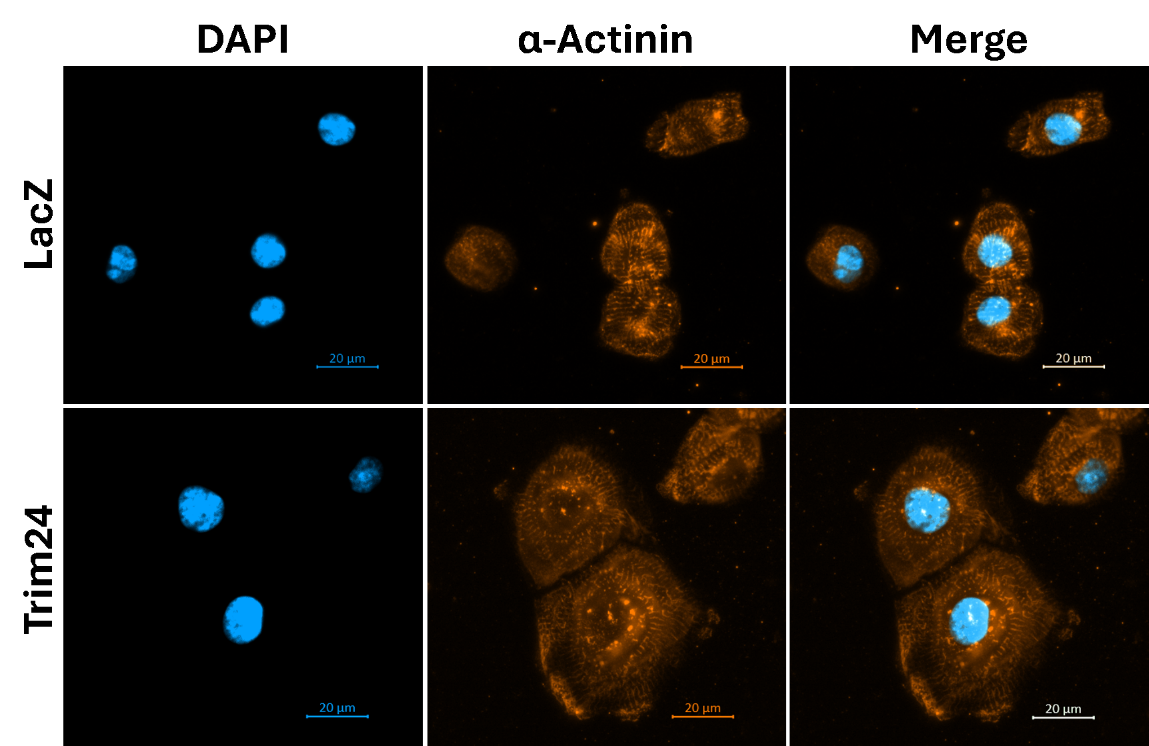


**Supplementary Figure 8: Trim24 overexpression induces cellular hypertrophy in human induced pluripotent stem cell-derived cardiomyocytes (hiPSC-CMs).** Representative images of hiPSC-CMs transduced with either LacZ (control) or Trim24. Cells were immunostained for α-actinin to visualize sarcomeric structure and counterstained with DAPI to label nuclei. Trim24 overexpression is associated with an increase in cell surface area compared to control. Scale bar: 20 µm.


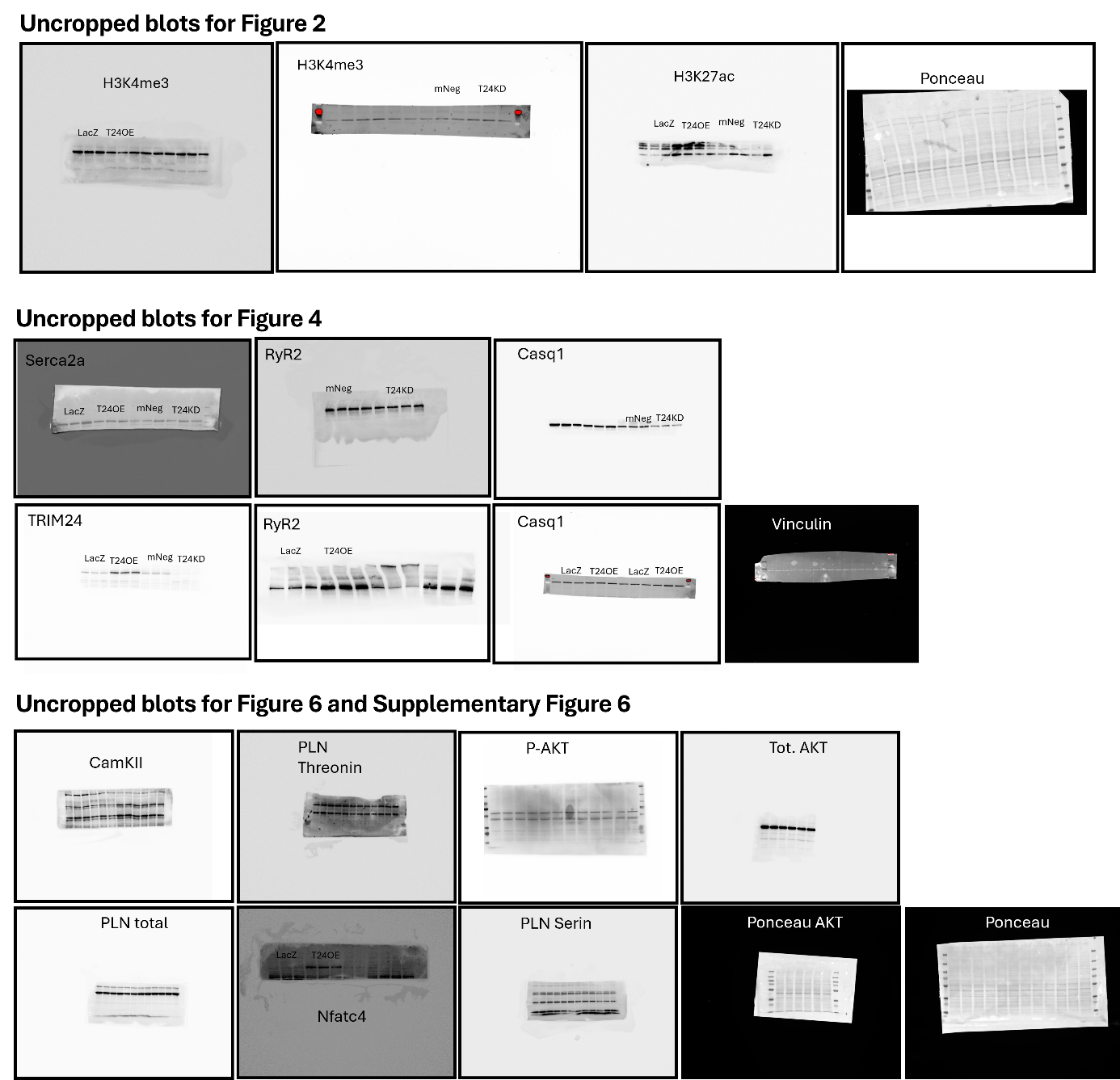


**Supplementary Figure 9: Uncropped blots presented in Figure 2, 4 and 5.**
