# Supplementary figures and images for: TRIM24 regulates chromatin remodeling and calcium dynamics in cardiomyocytes
Source: Cell Commun Signal. 2025 Jul 1;23:312. doi: 10.1186/s12964-025-02323-8 (PMC12211185; doi:10.1186/s12964-025-02323-8)

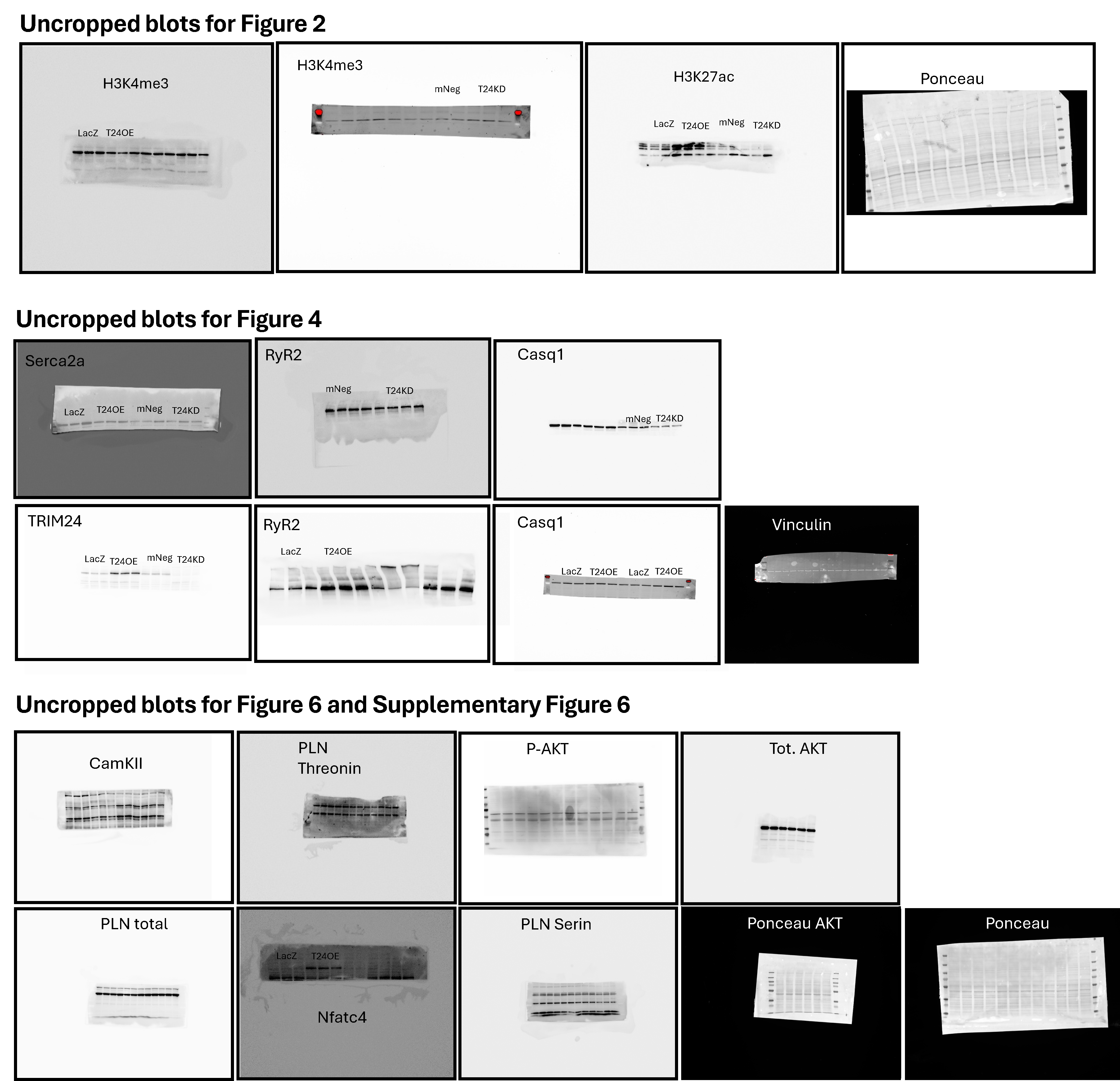

Supplement: Supplementary file 3 — Supplementary Material 3. [file 12964_2025_2323_MOESM3_ESM.png]
